# Glia-to-neuron transfer of miRNAs via extracellular vesicles: a new mechanism underlying inflammation-induced synaptic alterations

**DOI:** 10.1007/s00401-017-1803-x

**Published:** 2018-01-04

**Authors:** Ilaria Prada, Martina Gabrielli, Elena Turola, Alessia Iorio, Giulia D’Arrigo, Roberta Parolisi, Mariacristina De Luca, Marco Pacifici, Mattia Bastoni, Marta Lombardi, Giuseppe Legname, Dan Cojoc, Annalisa Buffo, Roberto Furlan, Francesca Peruzzi, Claudia Verderio

**Affiliations:** 1 CNR Institute of Neuroscience, via Vanvitelli 32, 20129 Milan, Italy; 2 Gastroenterology Unit, Department of Internal Medicine, University of Modena and Reggio Emilia, 41124 Modena, Italy; 3 Department of Neuroscience, Scuola Internazionale Superiore di Studi Avanzati (SISSA), via Bonomea 265, 34136 Trieste, Italy; 4 Department of Neuroscience Rita Levi-Montalcini and Neuroscience Institute Cavalieri Ottolenghi, University of Turin, 10126 Turin, Italy; 5 LSU Health Sciences Center School of Medicine and Stanley S. Scott Cancer Center, New Orleans, USA; 6 Clinical Neuroimmunology Unit, Department of Neuroscience, Institute for Experimental Neurology, San Raffaele Scientific Institute, via Olgettina 58, 20132 Milan, Italy; 7 IRCCS Humanitas, via Manzoni 56, 20089 Rozzano, Italy; 8 CNR-Institute of Materials, Area Science Park, 34149 Trieste, Basovizza Italy

**Keywords:** Microglia, Extracellular vesicles, miRNAs, Multiple sclerosis, Cognitive symptoms

## Abstract

Recent evidence indicates synaptic dysfunction as an early mechanism affected in neuroinflammatory diseases, such as multiple sclerosis, which are characterized by chronic microglia activation. However, the mode(s) of action of reactive microglia in causing synaptic defects are not fully understood. In this study, we show that inflammatory microglia produce extracellular vesicles (EVs) which are enriched in a set of miRNAs that regulate the expression of key synaptic proteins. Among them, miR-146a-5p, a microglia-specific miRNA not present in hippocampal neurons, controls the expression of presynaptic synaptotagmin1 (Syt1) and postsynaptic neuroligin1 (Nlg1), an adhesion protein which play a crucial role in dendritic spine formation and synaptic stability. Using a *Renilla*-based sensor, we provide formal proof that inflammatory EVs transfer their miR-146a-5p cargo to neuron. By western blot and immunofluorescence analysis we show that vesicular miR-146a-5p suppresses Syt1 and Nlg1 expression in receiving neurons. Microglia-to-neuron miR-146a-5p transfer and Syt1 and Nlg1 downregulation do not occur when EV–neuron contact is inhibited by cloaking vesicular phosphatidylserine residues and when neurons are exposed to EVs either depleted of miR-146a-5p, produced by pro-regenerative microglia, or storing inactive miR-146a-5p, produced by cells transfected with an anti-miR-146a-5p. Morphological analysis reveals that prolonged exposure to inflammatory EVs leads to significant decrease in dendritic spine density in hippocampal neurons in vivo and in primary culture, which is rescued in vitro by transfection of a miR-insensitive Nlg1 form. Dendritic spine loss is accompanied by a decrease in the density and strength of excitatory synapses, as indicated by reduced mEPSC frequency and amplitude. These findings link inflammatory microglia and enhanced EV production to loss of excitatory synapses, uncovering a previously unrecognized role for microglia-enriched miRNAs, released in association to EVs, in silencing of key synaptic genes.

**Electronic supplementary material:**

The online version of this article (10.1007/s00401-017-1803-x) contains supplementary material, which is available to authorized users.

## Introduction

Microglia, the resident immune cells of the brain, are fundamental to brain development and function [53], and have a recognized role in brain inflammation. Besides participating in information processing at the level of synapses, microglia are involved in complex brain functions, such as learning-induced synaptic plasticity, memory and cognition [49, 54, 55], and mouse behaviour [21, 44]. It is generally assumed that the broad activity of microglia relies on their capability to secrete soluble molecules, which not only mediate homeostatic synaptic plasticity in response to neuronal inputs [42, 54], but also cause synaptic damage upon early microglia responses [17]. Among microglia-derived soluble factors known to affect synaptic function and altering neuron excitability, inflammatory cytokines, i.e. TNF-α and IL-1β, play a critical role in chronic neuroinflammatory disorders, by modulating the expression and/or properties of synaptic channels [12, 55, 65, 79, 81], and regulating the expression of molecules critical for synaptic plasticity, including CREB or cofilin [73]. Recent studies have revealed that microglia can also regulate synapses through contact-dependent mechanisms, including engulfment of synaptic elements [74, 80, 82]. However, the mechanism(s) leading to microglia-mediated synapse loss during brain inflammation remain unknown and whether reactive microglia may disrupt synaptic structure and function by silencing synaptic genes has never been explored.

MicroRNAs (miRNAs) are small non-coding RNAs of approximately 22 nucleotides in length, known to regulate post-translational transcription [25]. The highest expression of tissue-specific miRNAs has been found in the brain [4, 67, 69], and a specific set of miRNAs are localized to dendrites, where local translation may affect dendritic spine morphology [68] and participate in adult neural plasticity [30, 46]. MiRNAs are abundant and play a key role also in the immune system [9, 83]. Once released by cells, miRNAs circulating in body fluids are promising markers of specific diseases, serving a diagnostic purpose particularly in brain “fluid biopsies” [36, 77]. Their suitability in diagnostics is also due to the remarkable stability of miRNAs to exogenous ribonucleases, being either associated to miRNA-processing molecules such as argonaute2 [2, 47], or packaged into EVs, which protect them from degradation [56].

EVs are membranous vesicles that bud from the plasma membrane (MVs/ectosomes) or result from exocytosis of multivesicular bodies (exosomes). Carrying a defined but mixed cargo of biomolecules, such as RNAs, proteins and lipids, EVs possess unique biological activities with the ability to modulate profoundly the molecular configuration and behaviour of target cells [15]. Recent evidence indicates that microglia and other glial cells can communicate with neurons through secretion of EVs [10, 43]. Microglia-derived EVs carry bioactive lipids, including endocannabinoids, which acutely impact neuronal firing rate [1, 29, 62]. Under pathological conditions, microglial EVs become vehicle of pathogenic proteins, e.g. the pro-inflammatory cytokine IL-1β [6], pathogenic Aβ and tau protein [3, 38] which are toxic to recipient neurons. EVs released by astrocytes contain and transfer miRNAs to metastatic tumour cells, thus promoting brain metastasis [86]. Importantly, the miRNA cargo of EVs produced by astrocytes and oligodendrocytes may also regulate the expression of neuronal genes [18, 27, 45]. Despite these advances, no formal proof for glia-to-neuron transfer of miRNAs has been provided, and whether vesicular miRNA is essential to alter gene expression in recipient neurons remains to be established.

In this study, we investigated whether inflammatory microglia transfer bioactive miRNAs to neurons through secretion of EVs. By exposing donor microglia to pro-inflammatory or pro-regenerative stimuli, we upregulated and downregulated, respectively, the level of miR-146a-5p in EVs and found that miR-146-5p-enriched EVs fuse with/and transfer their miR-146a-5p cargo to neurons, resulting in downregulation of miR-146a-5p synaptic targets with a considerable impact on synapse stability.

## Materials and methods

### Brain cell cultures

Mixed glial cell cultures, containing both astrocytes and microglia, were established from both male and female rat Sprague–Dawley pups (P2) (Charles River, Lecco, Italy) and maintained as previously described [59]. To obtain pure microglia (> 98%), cells were harvested from 10- to 14-day in vitro (DIV) cultures by orbital shaking for 30 min at 1300 r.p.m. and re-plated on poly-dl-ornithine-coated tissue culture dishes. The purity of the microglia preparation was identified by live staining with 0.01 mg/ml Isolectin-B4 Alexa 488-conjugated (Molecular Probes, Life Technologies Ltd., Paisley, UK) for 5 min at 37 °C, followed by staining with rabbit anti-GFAP or anti-NG2 and DAPI of fixed cells [4% paraformaldehyde–4% sucrose (w/v)] (Fig. S1). These cells express a partial molecular signature characteristic of acute ex vivo adult microglia [23]. Microglia were stimulated with a cocktail of Th1 cytokines (20 ng/ml IL-1β, 20 ng/ml TNF-α and 25 ng/ml IFN-γ), with 20 ng/ml IL-4 or 2 μM aggregated Aβ 1–42 for 24 h. Aβ 1–42 (Anaspec, Fremont, CA, USA) was dissolved at a concentration of 2 mM in DMSO, kept at − 80° and diluted in glial medium [38]. Successful polarization towards inflammatory or pro-regenerative phenotype under these conditions was confirmed (data not shown), according to previous studies [11, 78, 84].

Astrocytes were exposed to LPS 0.4 μg/ml for 6 h to induce an inflammatory response. Primary neuronal cultures were obtained from the hippocampi of 18-day-old foetal Sprague–Dawley rats (Charles River, Lecco, Italy). Briefly, dissociated cells were plated onto poly-l-lysine (Sigma Aldrich, Saint Louis, MO) treated coverslips/dishes and maintained in Neurobasal medium supplemented with 2% B27 supplement (Life Technologies, Carlsbad CA, USA). In a set of experiment neurons were treated with heparitinase I (0.5 mU/ml) for 30 min at 37 °C before treatment with EVs.

### Glia–neuron cocultures

To maintain neurons in co-culture with inflammatory microglia/astrocytes, neurons were grown on glass coverslips while microglia/astrocytes were plated separately on wells and treated with Th1 cytokines/LPS for 24 h. At the end of the incubation, cytokines/LPS were removed and neurons were introduced on top of glial cells, suspended over glial cells but not in contact with them, as established by Bartlett and Banker [5]. Every 24 h for a period of 72 h the coverslips with neurons were moved to a new well containing cytokine-primed microglia or LPS-primed astrocytes in order to avoid a change in the activation state of inflammatory cells. All the experimental procedures to establish primary cultures followed the guidelines established by the European legislation (Directive 2010/63/EU), and the Italian Legislation (LD no. 26/2014).

### Isolation, labelling and treatment of EVs

Cells were thoroughly washed and stimulated with 1 mM ATP for 30 min to favour MV secretion or for 1 h to induce accumulation of both MVs and exosomes in Krebs–Ringer’s HEPES solution (KRH) (125 mM NaCl, 5 mM KCl, 1.2 mM MgSO_4_, 1.2 mM KH_2_PO, 2 mM CaCl_2_, 6 mM d-glucose, and 25 mM HEPES/NaOH, pH 7.4). Conditioned KRH was collected and pre-cleared from cells and debris at 300×*g* for 10 min (twice). MVs were then pelleted from the supernatant by a centrifugation step at 10,000×*g* for 30 min, while exosomes were subsequently isolated from the supernatant at 100,000×*g* for 1 h. MVs- and exosome-enriched pellets were either lysed for RNA isolation using Direct-zol™ RNA MiniPrep kit (Zymo Research, Irvine CA, USA) or immediately re-suspended in neuronal medium. Unless otherwise specified, 1.7 × 10^5^ neurons were incubated with MVs derived from 2 × 10^6^ M1-/M2-microglia (1 × 10^8^ particle/ml) or 6 × 10^6^ astrocytes every 24 h for 72 h to keep EV concentration chronically elevated. In a set of experiments, MVs were treated with annexin-V (0.84 μg/ml) to cloak PS residues, before delivery to neurons by optical tweezers. For protection assay, MVs/exosomes have been incubated with 20 mU/ml RNase I and 1% TritonX-100 for 20 min at room temperature, as described previously [72].

### EV quantification

Tunable Resistive Pulse Sensing (TRPS) technique, by Izon qNano instrument (Izon, Christchurch, New Zealand), was used to measure the size distribution and concentration of particles in isolated MV- and exosome-enriched fractions. A reagent kit from Izon (Izon EV reagent kit) was used for both pre-treating the pore and suspending EVs in order to prevent EV binding to the pore or spontaneous EV aggregation. MVs or exosomes produced by 1 × 10^6^ microglia in 1 h were re-suspended in a volume of 100 μl. NP300 nanopore (150–600 nm diameter range; Izon) was used for MV sample analysis, while NP150 nanopore (85–300 nm diameter range; Izon) was used for exosome sample analysis. In each experiment, the same applied voltage, pressure and pore stretch values were set for all MV/exosome sample recordings and relative calibration. CPC200 calibration particles (carboxylated polystyrene particles, supplied by Izon and diluted following manufacturer’s instructions) were used as standards. They were measured immediately before or after the experimental samples under identical conditions. Data acquisition and analysis were performed using Izon Control Suite software (version V3.2). MV and exosome concentration values were normalized on protein concentration relative to donor cell sample, determined through bicinchoninic acid assay (BCA, Fisher Scientific, Waltham MA, USA).

### Exiqon miRNA profiling and data analysis

Total RNA was extracted from microglia, MVs and exosomes using the Direct-zol™ RNA MiniPrep kit (Zymo Research, Irvine CA, USA). For miRNA profiling, 25 ng of RNA from each sample was subjected to retro-transcription using the Universal cDNA synthesis kit (Exiqon, Woburn, MA), followed by qRT-PCR (SYBR green Master Mix, Exiqon). MiRNA arrays (V2.0 version) were carried out on a Roche Light Cycler 480 Real Time PCR system using the mouse/rat panel I. Raw data were converted into cycle threshold (*C*_t_) measurements by the Roche Light Cycler system software (Version 1.5; Roche). Quantification using 2nd derivative maximum was further calculated with Roche Light Cycler 480 software. Q-PCR data were further analysed in GenEx Professional 5 software (MultiD Analyses AB, Goteborg, Sweden). After inter-plate calibration, a cut-off of 38 was applied and the amount of target miRNAs was normalized relative to the amount of RNU1A1 and RNU5G, as determined by geNorm application incorporated into GenEx. Relative quantification was calculated as fold-change of miRNA expression in the experimental groups according to the formula 2^−ΔΔ*C*t^, as previously [24, 41, 51, 52].

### Quantitative real-time PCR

q-PCR for detection of mature miR-146a-5p, miR-181a and miR-223 microRNAs was performed using the miRCURY LNA™ Universal RT microRNA PCR system (Exiqon) according to the manufacturer’s instructions. The assays were run on a QuantStudio™ 5 (ThermoFisher Scientific, Waltham MA, USA) real-time PCR system. Relative quantification was calculated using the QuantStudio™ 5 design and analysis software (Applied Biosystems, Foster City CA, USA) (based on the 2nd derivative maximum). In the RNase I protection assay, where the endogenous control (RNU1A1) is degraded as well as the miRNAs of interest as consequence of RNase treatment, the relative abundance of miRNAs was calculated from the expression 2^ − (*C*_texperimental_ − *C*_tcontrol_), expressed in percentage [72]. The miRNA contents of human CSF EVs and of cultured neurons were analysed independently of any other sample, therefore the relative abundance was expressed as d*C*_t_: *C*_ttarget_ − *C*_treference_, where RNU1A1 is considered as the endogenous control.

**Table:** 

Exiqon primers	Cat. number
rno-miR-181a	Ref# 206081
rno-miR-146a-5p	Ref# 204688
mmu-miR-223	Ref# 205986
mmu-has-RNU1A1	Ref# EX203909

### CSF samples

Human CSF has been obtained for diagnostic purposes from subjects with multiple sclerosis according to revised McDonald criteria [58] attending the MS Center at San Raffaele Hospital (Milan, Italy). Total EVs were isolated from samples smaller than 1 ml of CSF pooled together from ten MS patients. MiRNAs were isolated and q-PCR was performed as described above. This research project was approved by the ethical committee of San Raffaele Scientific Institute, and all subjects gave written informed consent.

### Dual luciferase assay

The mature sequences of miR-146a-5p and cel-miR-39 were cloned into BlockIt (Invitrogen) following the manufacturer’s protocol, as described previously [64]. For the cloning of the perfect match sequence into pSiCheck-2 vector (Promega, Madison, WI, USA), the PCR primers were based on the mature sequence of miR-146a-5p/cel-miR-39 but in the antisense orientation.

Neurons were transfected with a total amount of 0.3 μg/well of DNA (miR-146a-5p pSiCheck; cel-miR-39 pSiCheck) using Lipofectamine 2000™ (Invitrogen), at DIV 7–8, when higher transfection efficiency is achieved compared to mature neurons. After 24 h 1.7 × 10^5^ transfected neurons were incubated overnight with glial MVs secreted by 1 × 10^6^ microglia (2.3 × 10^7^ particle count/ml) or 3 × 10^6^ astrocytes, which release less EVs compared to microglia [1] in 30–60 min. Samples were harvested 48 h post-transfection and subjected to the dual luciferase assay system (Promega). Donor astrocytes were transfected with anti-miR-146a-5p (30 nM miRCURY LNA; Exiqon) using Lipofectamine 2000 before EV isolation. To verify miR-146a-5p inactivation astrocytes were co-transfected with miR-146a-5p pSiCheck and anti-miR-146a-5p. In a set of experiments EVs were treated with annexin-V (0.84 μg/ml) for 30 min at room temperature, before being applied to neurons.

### Confocal analysis of Syt1 immunoreactivity

Hippocampal neurons were transfected at DIV 7–8 with eGFP or miR-146a-5p (BlockIt) (1 μg of DNA/coverslip) using Lipofectamine 2000. After 24 h 1.7 × 10^5^ neurons were incubated with MVs derived from 6 × 10^6^ astrocytes for 48 h, fixed with 4% paraformaldehyde–4% sucrose (w/v) and stained with rabbit anti-Syt1 and goat anti-GFP FITC conjugated, followed by Alexa-555 conjugated secondary antibodies (1:200, Alexa-Invitrogen, San Diego, CA). Images were acquired using a Leica SPE confocal microscope equipped with a 63 × /1.30 NA oil objective (Leica Microsystems, Solms, Germany). Acquisition parameters (i.e. laser power, gain and offset) were kept constant among different experimental settings. Syt1 staining was quantified by the ImageJ software (http://rsb.info.nih.gov/ij/) on Z-stacks, in order to measure the signal coming from the total volume of the cells. ROIs were drawn on the somatodendritic compartment and the fluorescence intensity for Syt1 was measured with the histogram function of the software after definition of an arbitrary, but homogeneous threshold. The same was done for the eGFP signal, which was saturated purposely, giving a measure of the total number of pixel present in the volume. Syt1 expression has been expressed as the fluorescence intensity normalized for the cell body volume.

### Western blotting

Neurons treated with EVs for 72 h were lysed with a buffer containing 1% sodium dodecyl sulfate (SDS), 10 mM HEPES, 2 mM EDTA pH 7.4. A modified version of the Laemmli buffer (20 mM Tris pH 6.8, 2 mM EDTA, 2% SDS, 10% glycerol, 2% β-mercaptoethanol, 0.01% bromophenol blue) was then added to a final 1× concentration and proteins were separated by gel electrophoresis, blotted on nitrocellulose membrane filters and probed using the antibodies reported in the table. Photographic development was by chemiluminescence (ECL, GE Healthcare) according to the manufacturer’s instructions. Western blot bands were quantified by ImageJ software.

**Table:** 

Antibody	Host	Supplier	WB dilution	IF dilution
Anti-β-III tubulin	Mouse	Promega (Madison, WI, USA)	1:4000	
Anti-Bassoon	Guinea pig	Synaptic Systems (Goettingen, Germany)		1:500
Anti-GAPDH	Rabbit	Synaptic Systems (Goettingen, Germany)	1:2000	
Anti-GFAP	Rabbit	Synaptic Systems (Goettingen, Germany)		1:300
Anti-GFP-FITC	Goat	Novus Biologicals (Littleton, CO, USA)		1:300
Anti-MAP2	Mouse	Sigma Aldrich (Saint Louis, MO, USA)	1:1000	
Anti-NG2	Rabbit	Millipore (Burlington, MA, USA)		1:100
Anti-Nlg1	Mouse	Synaptic Systems (Goettingen, Germany)	1:1000	
Anti-NR2B	Mouse	Synaptic Systems (Goettingen, Germany)	1:1000	
Anti-PSD95	Mouse	NeuroMab (Davis, CA)	1:5000	
Anti Shank2	Rabbit	Synaptic Systems (Goettingen, Germany)		1:500
Anti-SNAP25	Mouse	SMI81 Sternberger Monoclonals	1:10000	
Anti-Syt1	Rabbit	Synaptic Systems (Goettingen, Germany)	1:1000	1:200

### Cell viability assays

The assay was performed as previously described [38]. Neuron viability was analysed by simultaneous fluorescence staining of viable and dead cells with calcein-AM (0.5 mg/ml, Invitrogen, Life Technologies Ltd.), propidium iodide (1 mg/ml, Molecular Probes, Life Technologies Ltd., Paisley, UK) and Hoechst (8.1 mM, Molecular Probes, Life Technologies Ltd.). Incubation was performed for 20 min in neuronal medium at 37 °C and 5% CO_2_. Calcein-AM emits green fluorescence signal in viable cells, while PI reaches nuclei of only dead cells emitting red fluorescence. Fluorescence images were acquired by Leica DMI 4000B microscope (Leica Microsystems GmbH, Wetzlar, Germany), equipped with DIC microscopy. The percentage of neuronal death was calculated as the ratio of PI^+^/calcein^+^-dead cells to the total number of Hoechst-stained neurons in at least 15 fields/condition.

### RFP-transfection and dendritic spine analysis

1.7 × 10^5^ neurons were transfected at DIV14 with a RFP or an Nlg1-HA expressing vector [pCAG-NL1(−)], gift from Peter Scheiffele (Addgene plasmid #15260) using Lipofectamine 2000 (0.5 μg of plasmid). At this developmental stage the efficiency of transfection is quite low (~ 5%). Neurons were then incubated with MVs for 72 h, fixed and stained with guinea pig anti-Bassoon and rabbit anti-Shank2. Secondary antibodies were conjugated with Alexa-488 and Alexa-633 (1:200, Alexa-Invitrogen, San Diego, CA). Neurons were imaged with a 63× objective using an Axiovert 200 M (Zeiss) confocal system equipped with a spinning disk (UltraVIEW acquisition system, Perkin Elmer) keeping acquisition parameters constant. Focal planes were stacked together in a projection, and RFP-positive spines were counted on 20–40 μm segments of secondary dendrites. The spine density was calculated as number of spines per 10 μm of dendrite. The abundance of different types of spines (mushroom, thin and stubby) was analysed using the ImageJ software (http://imagej.nih.gov/ij/). Spines were classified in categories based on morphological parameters: spine head diameter (H), spine length (L) and spine neck width (N), according to NeuronStudio software criteria: mushroom spines: H/N > 1.1 μm and H > 0.35 μm; thin spines: H/N > 1.1 μm and H < 0.35 μm or H/N < 1.1 μm and L/H > 2.5 μm; stubby spines: H/N < 1.1 μm and L/H < 2.5 μm. Approximately 60 spines per field were measured in at least 15 dendrites per independent experiments.

The percentage of synapses with pre- and post-synaptic terminals was measured in a single confocal plane as follows: Bassoon and Shank2 double-positive puncta were revealed by generating a Bassoon/Shank2 double-positive image using the ‘and’ option of ‘image calculator’ function. A fixed threshold was then set in the double-positive image and number of double-positive puncta was quantified using the ‘analyze particle’ function and normalized to the total number of Shank2-positive puncta to obtain the fraction of Shank2-positive presynaptic terminals.

### Electrophysiological recordings

Non-transfected hippocampal neurons were incubated with MVs for 72 h. Whole-cell voltage clamp recordings were then performed using a MultiClamp 700A amplifier (Molecular Devices) coupled to a pCLAMP 10 Software (Molecular Devices), and using an inverted Axiovert 200 microscope (Zeiss). MEPSC were recorded from DIV 13–15 neurons using external control solution KRH, in the presence of 1 µM TTX. Experiments were performed at room temperature (20–25 °C), setting the holding potential at − 70 mV and using the following internal solution: 130 mM CsGluc, 8 mM CsCl, 2 mM NaCl, 10 mM HEPES, 4 mM EGTA, 4 mM MgATP, 0,3 mM Tris-GTP (pH 7.3, adjusted with CsOH). Traces were sampled at 10 kHz and filtered at 2 kHz. Series resistance was monitored during recording. mEPSC were detected using Clampfit software (Molecular Devices) setting a threshold of 5 pA. The mean mEPSC frequency for CTRL neurons was 1.537 ± 0.356 Hz (mean ± SE), their mean amplitude was 25.445 ± 1.503 pA (mean ± SE).

### In vivo EVs delivery and dendritic spine analysis

In vivo experiments were performed on 4-month-old C57BL6 male mice (*n* = 3/condition). Surgery and perfusions were carried out under deep general anAesthesia (ketamine, 100 mg/kg; Ketavet, Bayern, Leverkusen, Germany; xylazine, 5 mg/kg; Rompun, Bayer, Leverkusen, Germany). The experimental plan was conducted in accordance with the European directive (2010/63/EU) and the Italian Law for Care and Use of Experimental Animals (DL116/92). It was also approved by the Italian Ministry of Health and the Bioethical Committee of the University of Turin. Osmotic minipumps (Alzet osmotic pumps 1007D) were implanted unilaterally in CA1 (anteroposterior: 1.8 mm relative to bregma; mediolateral: +1 mm; dorsoventral: 1.5 mm relative to the dura) where they infused rat M1-EVs (1.5 µg/µl, diluted in sterile saline), M2-EVs or saline (vehicle) at 0.5 µl/h for 4 days. The contralateral side was used as additional control (intact). The brains were removed and stained by modified Golgi–Cox method as described in [85]. Coronal sections of 100 µm thickness from the dorsal hippocampus were obtained using a vibratome (Leica Microsystems GmbH). Slices were mounted on gelatin-coated microscope slides, stained, dehydrated and mounted with a xylene-based medium. Images of Golgi sections were acquired through Video-Confocal Microscope (VICO, Nikon Instruments), with a 60× oil immersion lens. Spine density was quantified on the secondary branches of neurons located in the CA1 of the dorsal hippocampus, by ImageJ software. At the site of minipump implantation dendrites were analysed at distances ranging from 50 to 400 μm from the cannula track, to exclude artefacts due to direct neuron damage.

### Optical tweezers

An IR laser beam (1064 nm, CW) for trapping was coupled into the optical path of an inverted microscope (Axiovert 200 M, Zeiss) through the right port of the microscope. The trapping beam was directed to the microscope lens (Zeiss 63X, NA 1.4) by the corresponding port mirror (100%) and the tube lens. Optical trapping and manipulation of EVs was performed following the approach previously described [59]. Immediately before recording, MVs produced by glial cells pre-loaded with calcein/AM (20 nM for 45 min) were added to neurons plated on glass coverslips and maintained in 400 µl of neuronal medium in a temperature controlled recording chamber at 37 °C. As soon as a calcein-positive MV appeared in the recording field, it was trapped and positioned on a selected neuron by moving the cell stage horizontally and the microscope lens axially. After about 30 s from contact, the laser was switched off to prove EV–neuron interaction. During the experiments neurons were live imaged with a spinning disk confocal microscope (UltraVIEW acquisition system, Perkin Elmer Waltham, MA, USA) using a digital camera (High Sensitivity USB 3.0 CMOS Camera 1280 × 1024 Global Shutter Monochrome Sensor, Thorlabs, Newton, NJ, USA) at a frame rate of 2 Hz.

### Membrane fusion assay

In another set of experiments, MVs have been obtained from glial cells (1 × 10^6^) preloaded with calcein-AM (0.5 mg/ml) for 20 min in complete medium at 37 °C and 5% CO_2_. Calcein-AM loaded EVs were then labelled with the self-quenching lipophilic dye octadecyl rhodamine B chloride (R18, 0.2 µM) (Life Technologies, Carlsbad CA, USA) for 30 min at room temperature, washed twice in KRH and re-pelleted at 10,000×*g* before being added to 1.7 × 10^5^ neurons and live imaged. Fluorescence of calcein-R18 doubled labelled MVs approaching the surface of neurons in the observation field was monitored at 510–590 nm emission for 5 min. Images were acquired at 2 Hz. Calcein/R18 mean fluorescence intensity was measured using ImageJ software at ROIs selected around MVs in sequence of images to obtain temporal analysis. Fluorescence changes were expressed as *F*–*F*_0_, where *F*_0_ is the fluorescence measured when the MV enters the focal plane.

### Statistical analysis

All data are presented as mean ± SE from the indicated number of independent experiments. Statistical analysis was performed using SigmaPlot 12.0 (Jandel Scientific, San Jose, CA, USA) software. After testing data for normal distribution, the appropriate statistical test has been used (see figure legends). Differences were considered significant when *P* was < 0.05, *P* < 0.01 and *P* < 0.001 and they are indicated by one, two or three asterisks, respectively.

## Results

### EVs contain a selected set of miRNAs of parental microglia

**Fig. 1:**
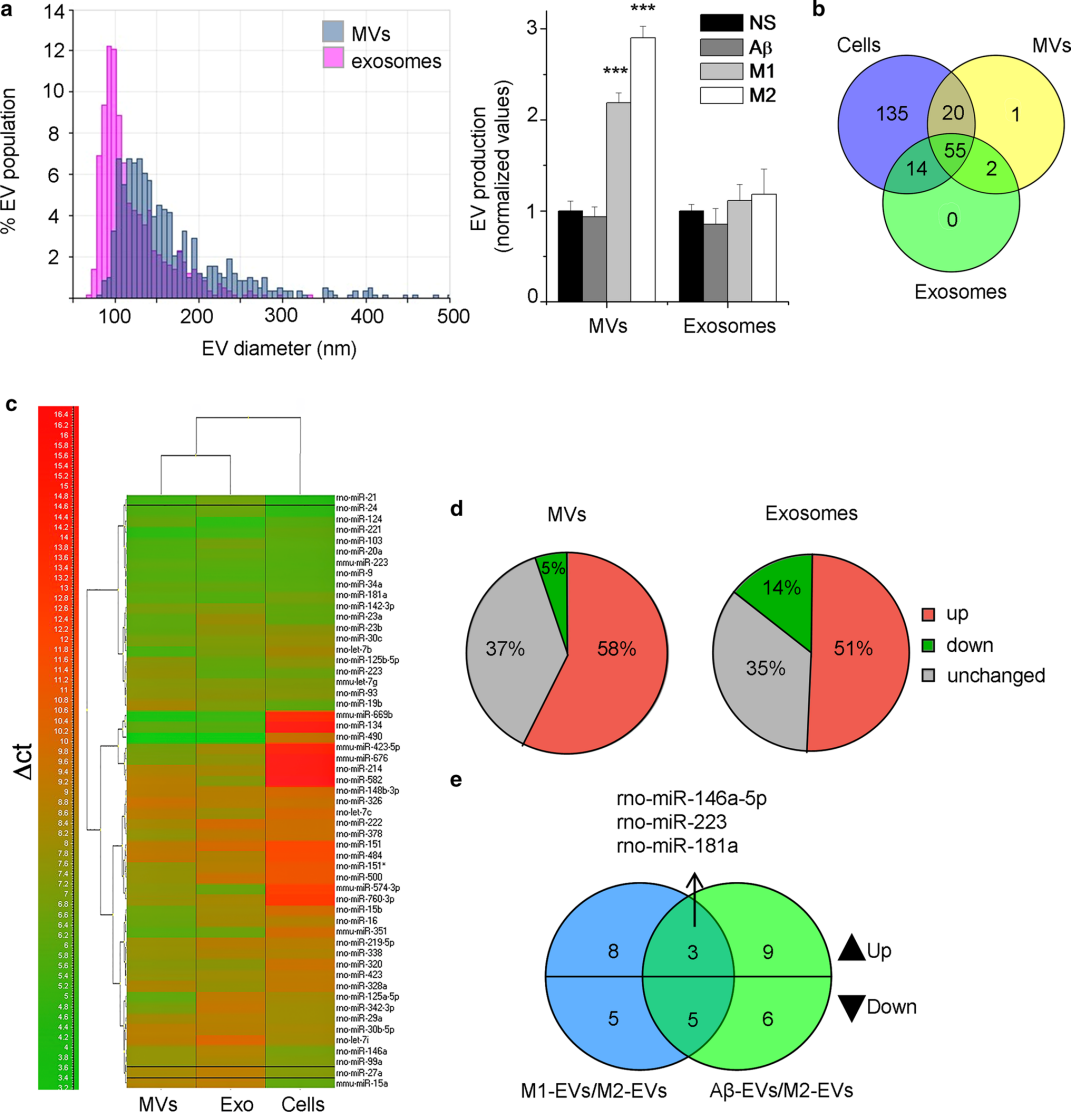
Analysis of miRNAs in EVs released from microglia. **a** Size profile of MVs and exosomes pelleted from 1 × 10^6^ NS microglia, re-suspended in 100 μl of 0.1 µm-filtered PBS and analysed using TRPS (left). Histograms show production of MVs and exosomes from microglia polarized with different agents during 1 h stimulation with ATP. Data are normalized to unstimulated condition (MVs, one-way ANOVA *P* ≤ 0.001; Holm–Sidak test versus control; exosomes: one-way ANOVA *P* = 0.501). **b** Venn diagram of the numerical values for common and unique miRNAs present in MVs (yellow), exosomes (green) and parental microglia under resting condition (blue). **c** Heat map of ∆*C*_t_ of miRNAs expressed in MVs, exosomes and unstimulated cells. Red indicates low expression and green indicates high expression. **d** Pie charts depict the percentage of miRNAs that are upregulated (red), downregulated (green) and unchanged (grey) in MVs or exosomes versus unstimulated microglia. **e** Venn diagram of EV miRNAs differentially expressed in M1-EVs and Aβ-EVs versus M2-EVs

Newborn rat microglia were maintained in culture medium enriched with inflammatory (Th1 cytokines), degenerative (amyloidogenic peptide Aβ 1–42) or pro-regenerative (IL-4) stimuli. Polarized cells were then exposed to ATP for 1 h to promote release of MVs but also accumulation of exosomes. MVs and exosomes were isolated by differential centrifugation as described [29]. Our previous evidence indicates that EVs isolated under short ATP stimulation are negative for apoptotic markers and are not contaminated by intracellular organelles derived from damaged cells, as indicated by electron microscopy [6, 78] and western blot analysis [29]. Quantification by tunable resistive pulse sensing (TRPS) revealed that unstimulated microglia (1 × 10^6^ cells) showed secretion rate of ~ 3.5 × 10^7^ MVs (mean diameter = 182.82 ± 3.35 nm) and ~ 1 × 10^8^ exosomes (mean diameter = 92 ± 5.28 nm) per hour. Pro-inflammatory and pro-regenerative microglia produce more MVs compared to unstimulated cells, with no significant changes in exosome production (Fig. 1a).

MiRNA profiling in total RNA from MVs, exosomes and parental cells using a comprehensive mouse and rat miRNA panel and RNU1A1 and RNU5G as normalizers, revealed 245 miRNAs in at least two out of the three samples, which were then selected for further analysis. We found a total of 78 and 71 miRNAs, respectively, in MVs and exosomes produced by non-stimulated (NS) microglia, of which 21 were unique in MVs and 14 unique in exosomes (Fig. 1b), with a strong overlap between the two EV populations (57 common miRNAs). This overlap may derive in part from the centrifugation procedure used to collect EVs, which does not allow a precise separation of the two EV populations. Compared to parental microglia, EVs contained a selection of miRNAs and few vesicle-specific miRNAs, not detectable in donor cells. Q-PCR analysis showed that some miRNAs were several folds more represented in MVs and exosomes than in donor cells, while others were low abundant in MVs and exosomes than in cells (Fig. 1c, Fig. S2). This suggested that vesicular miRNAs are not the result of random sampling. A similar sorting mechanism appeared to regulate miRNA traffic to EVs produced by reactive microglia (Fig. S3a–c). Relative abundance of EV miRNAs versus cellular miRNAs showed that more than 50% of miRNAs were upregulated (fold change > 2) in MVs and exosomes produced by NS microglia (Fig. 1d).

### Identification of miRNAs altered in EVs produced by pro-inflammatory versus pro-regenerative microglia

**Fig. 2:**
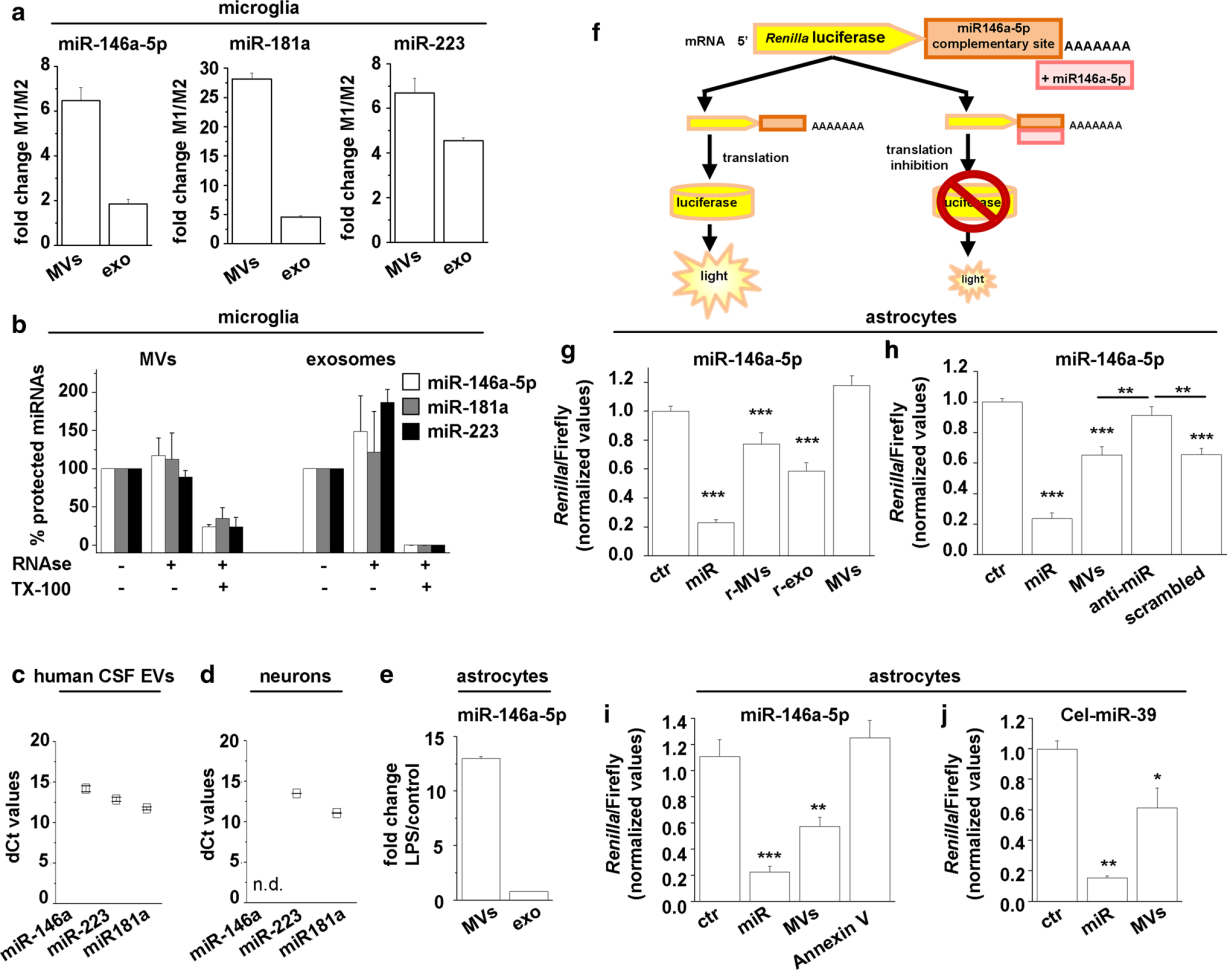
Glia-to-neuron shuttling of miR-146a-5p via MVs. **a** Histograms show relative quantification of miR-146a-5p miR-181a and miR-223 in MVs and exosomes released from inflammatory (M1) versus pro-regenerative (M2) microglia. **b** RNase I protection of miR-146a-5p, miR-181a and miR-223 quantified by q-PCR in MVs and exosomes produced by M1 microglia. EVs were treated with or without RNase I and/or Triton X-100 (*n* = 2). **c** Graph shows dCt values of miR-146a-5p, miR-181a and miR-223 in EVs derived from human CSF from 10 MS patients. **d** Graph shows dCt values of miR-146a-5p (undetectable), miR-181a and miR-223 in cultured neurons. **e** Representative q-PCR showing increased miR-146a-5p expression in MVs and exosomes released from LPS-treated versus control astrocytes. **f**–**j** 7- to 8-day-old neurons were transfected with miR-146a-5p or cel-miR-39 reporter, incubated overnight with EVs, harvested and subjected to the dual luciferase assay system using a multiwell plate reader. **f** Schematic representation of the *Renilla/*firefly-based reporter for miR-146a-5p. **g** The histogram shows measures of *Renilla*/firefly activity in control untreated neurons (ctr), neurons co-transfected with 0.7 μg/ml miR-146a-5p (miR), and neurons exposed overnight to MVs or exosomes derived from reactive astrocytes (r-MVs, r-exo) or unstimulated cells (MVs). Relative units represent ratio between *Renilla* values and luciferase internal control. (ANOVA *P* < 0.0001 Holm–Sidak multi-comparison test: **P < 0.01, ***P < 0.001, *n* = 5). **h** *Renilla*/firefly luciferase assay in neurons exposed to MVs derived from astrocytes treated with anti-miR-146a-5p inhibitor (anti-miR) or a non-targeting sequence (scrambled). ANOVA *P* < 0.001 Holm–Sidak multi-comparison test: **P < 0.01, ***P < 0.001, *n* = 3. **i** *Renilla*/firefly luciferase assay in neurons exposed to MVs pretreated or not with annexin-V (Annexin V), to limit MV–neuron contact. Kruskal–Wallis *P* < 0.001 Dunn’s multi-comparison test **P < 0.01, ***P < 0.001, *n* = 3. **j** Donor astrocytes were transfected cel-miR-39, MVs pelleted from the supernatant and exposed to neurons transfected with a specific miR-39 reporter, before *Renilla* and luciferase detection. ANOVA *P* < 0.0001 Holm–Sidak multi-comparison test: *P < 0.05 **P < 0.01, *n* = 3

We next analysed miRNAs differentially expressed in EVs (MVs or exosomes) released from microglia stimulated with inflammatory cytokines (M1-EVs), Aβ 1–42 (Aβ-EVs) or with IL-4 (M2-EVs). Differential expression analysis of M1-EVs versus M2-EVs revealed 21 miRNAs, among which 11 were upregulated and 10 downregulated in M1-EVs (Fig. 1e; Table 1). Similarly, 12 upregulated and 11 downregulated miRNAs were differentially expressed between Aβ-EVs and M2-EVs (Fig. 1e; Table 2). We then searched for miRNAs upregulated in both M1-EVs and Aβ-EVs. We found three miRNAs that have validated dendritic targets, namely rno-miR-146a-5p, rno-miR-181a and rno-miR-223 (Fig. 1e; Tables 1, 2). MiR-146a-5p targets the postsynaptic cell-adhesion molecule neuroligin1 (Nlg1), the dendritic protein MAP1A and the synaptic vesicle protein synaptotagmin1 (Syt1) [39]; miR-181a targets the AMPA-selective glutamate receptor 2 (GluR2) [66]; while miR-223 targets both GluR2 and the NMDA glutamate receptor GluN2B [33]. All the synaptic targets of miR-146a-5p, miR-181a and miR-223 are conserved between mouse and rat and Syt1 is a conserved target also in human, as documented by Saba and coworkers [66] and the miRNA databases TargetScan v 7.1 and microRNA.org. Q-PCR analysis confirmed upregulation of miR-146a-5p, miR-181a and miR-223 in EVs released from inflammatory microglia, especially in shed MVs, compared to pro-regenerative cells (Fig. 2a). As expected for miRNAs packaged into a membrane bound compartment, they were protected from degradation by RNase I unless a detergent was added to disrupt the membrane of EVs (Fig. 2b).

**Table 1:** List of miRNAs upregulated or downregulated in MVs (normal font) and exosomes (italic font) produced from inflammatory (M1) microglia versus pro-regenerative (M2) cells

M1-EVs vs M2-EVs			
miRNAs upregulated	Fold change	miRNAs downregulated	Fold change
**rno-miR-181a**	2.34	rno-miR-490	0.26
rno-miR-30c	2.46	rno-miR-124	0.35
**mmu-miR-223**	2.85	rno-miR-134	0.42
**rno-miR-146a**	6.92	mmu-miR-297b-3p	0.45
*mmu-let-7g*	*3.27*	*mmu-miR-669b*	*0.23*
*mmu-miR-15a*	*3.01*	*mmu-miR-676*	*0.36*
*rno-miR-125a-5p*	*2.14*	*rno-miR-150*	*0.36*
***rno-miR-146a***	*5.81*	*rno-miR-214*	*0.25*
*rno-miR-16*	*2.56*	*rno-miR-290*	*0.17*
*rno-miR-17-1-3p*	*2.79*	*rno-miR-582*	*0.16*
*rno-miR-219-5p*	*2.05*		
*rno-miR-23a*	*12.38*		

Normal font denotes MVs-associated miRNAs

Italic font denotes exosomes-associated miRNAs

Bold font denotes miRNAs upregulated in both M1-EVs vs M2-EVs (this Table) and Abeta-EVs vs M2-EVs (Table 2)

**Table 2:** List of miRNAs upregulated or downregulated in MVs (normal font) and exosomes (italic font) produced from Aβ-treated microglia versus pro-regenerative cells

Aβ-EVs vs M2-EVs			
miRNAs upregulated	Fold change	miRNAs downregulated	Fold change
**mmu-miR-223**	32.33	*mmu-miR-297b-3p*	*0.28*
mmu-miR-345-3p	2.72	*mmu-miR-351*	*0.34*
mmu-miR-582-3p	3.85	*mmu-miR-574-3p*	*0.48*
mmu-miR-676	2.88	*mmu-miR-669b*	*0.20*
rno-miR-142-3p	5.40	*mmu-miR-676*	*0.43*
**rno-miR-146a**	22.70	*rno-let-7c*	*0.37*
**rno-miR-181a**	4.58	*rno-let-7i*	*0.17*
rno-miR-19b	3.06	*rno-miR-290*	*0.45*
rno-miR-20a	5.19	*rno-miR-338*	*0.36*
rno-miR-345-5p	17.09	*rno-miR-490*	*0.32*
rno-miR-34a	4.39	*rno-miR-582*	*0.29*
rno-miR-99a	4.13		
***rno-miR-146a***	*4.39*		

Normal font denotes MVs-associated miRNAs

Italic font denotes exosomes-associated miRNAs

Bold font denotes miRNAs upregulated in both Abeta-EVs vs M2-EVs (this Table) and M1-EVs vs M2-EVs (Table 1)

### miR-146a-5p, miR-181a and miR-223 are present in EVs of patients with multiple sclerosis

Previous evidence indicates that miR-146a-5p, miR-181a and miR-223 are dysregulated in patients affected by neuroinflammatory [36] and neurodegenerative diseases [22, 37, 60], that are characterized by high production of myeloid EVs [38, 78]. In particular, consolidated evidence describes miR-146a-5p upregulation both at MS lesions [40] and in CSF from patients with Alzheimer’s diseases [32, 61]. To validate the presence of the three miRNAs in EVs produced by reactive microglia in vivo, we isolated a mixed population of EVs (MVs and exosomes) from a pool of CSF collected from 10 patients affected by multiple sclerosis (MS). Q-PCR analysis confirmed the presence of miR-146a-5p, miR-181a and miR-223 in the EVs isolated from CSF, where EVs of myeloid origin accounts for ~ 65% of total EVs detectable by flow cytometry [38] (Fig. 2c).

### miR-146a-5p expression in astrocyte-derived EVs but not in hippocampal neurons in vitro

Among the three miRNAs upregulated in EVs produced by inflammatory microglia, miR-146a-5p is a microglia-enriched miRNA, detectable at lesser extend in astrocytes, and it prevents the expression of neuronal genes in neuron progenitors [39]. Thus miR-146a-5p appeared as an ideal candidate to investigate possible glia-to-neuron shuttling of miRNAs through EVs. Q-PCR analysis confirmed that miR-146a-5p is present in cultured astrocytes and MVs thereof, more abundantly upon activation with LPS (Fig. 2e), although astrocytes express less miR-146a-5p compared to LPS-treated microglia (fold change microglia versus astrocytes: 73.96 ± 5.67). Conversely, miR-146a-5p was undetectable in cultured hippocampal neurons unlike miR-181a and miR-223 (Fig. 2d). We next explored possible miR-146a-5p shuttling from EVs to neurons by dual luciferase assay using astrocyte-derived EVs in addition to microglial EVs, as astrocytes are more amenable of transfection than microglia, making easier the manipulation of their miR-146a-5p expression.

### EVs secreted from inflammatory glia transfer miR-146a-5p to neurons

Hippocampal neurons were transfected with a *Renilla*-based miR-146a-5p reporter and exposed overnight to EVs, either MVs or exosomes, derived from inflammatory astrocytes. The reporter carries *Renilla* gene fused to miR-146a-5p complementary sequence and firefly luciferase gene as normalization control. MiR-146a-5p binding to its match sequence inhibits translation of *Renilla* mRNA and decreases *Renilla* light emission (miR-146a-5p pSiCheck) [64] (Fig. 2f). Overnight exposure to reactive EVs (r-MVs or r-exosomes), but not MVs derived from unstimulated astrocytes (MVs), caused a significant reduction in *Renilla*/firefly luciferase activity in EV-treated neurons compared to control untreated neurons, indicating an increase in neuronal miR-146a-5p levels (Fig. 2g). By contrast, MVs released from astrocytes transfected with anti-miR-146a-5p (30 nM miRCURY LNA) did not reduce activity (Fig. 2h). MiR-146a-5p inactivation in donor astrocytes was indicated by enhanced *Renilla/*firefly luciferase activity in astrocytes transfected with the reporter (normalized *Renilla*/firefly luciferase activity: anti-miR-146a-5p = 1.971 ± 0.249, scramble = 1.167 ± 0.169; Student’s *t* test, *P* = 0.037; *n* = 4) while spectrometric quantification of MVs, performed according to [6], excluded alterations in MV production in anti-miR-146a-5p-treated astrocytes (Fluorescence intensity: controls = 30.000 ± 2.061, anti-miR-146a-5p = 33.885 ± 2.091, scramble 29.353 ± 2.240). Finally, the EV–neuron adhesion blocker annexin-V (0.84 μg/ml, see below Fig. 7b) prevented the *Renilla*/firefly activity reduction induced by MVs (Fig. 2i). MVs or exosomes secreted from inflammatory microglia caused a similar reduction in *Renilla*/firefly luciferase activity (normalized *Renilla*/Firefly luciferase activity: control = 1 ± 0.06; MVs = 0.62 ± 0.04; exosomes = 0.61 ± 0.04; *n* = 4, ANOVA *P* ≤ 0.001; Holm–Sidak multi-comparison test: MVs vs. control *P* < 0.0001). Taken together these data strongly suggested that miR-146a-5p-enriched EVs, secreted from inflammatory astrocytes or microglia, transfer their miR-146a-5p cargo to neurons.

To obtain definitive proof for glia-to-neuron shuttling of miRNAs via MVs we monitored neuronal levels of cel-miR-39, a *C. elegans* miRNA, not expressed in rat cells, which cannot be upregulated in neurons upon contact with MVs. Neurons were transfected with a *Renilla*-based cel-miR-39 reporter and incubated with MVs released from astrocytes exogenously expressing the miRNA. MVs released from cel-miR-39-transfected astrocytes efficiently transferred cel-miR-39 into neurons as indicated by reduced *Renilla/*firefly luciferase activity (Fig. 2j).

### Vesicular miR-146a-5p regulates Syt1 and Nlg1 protein expression in neurons

**Fig. 3:**
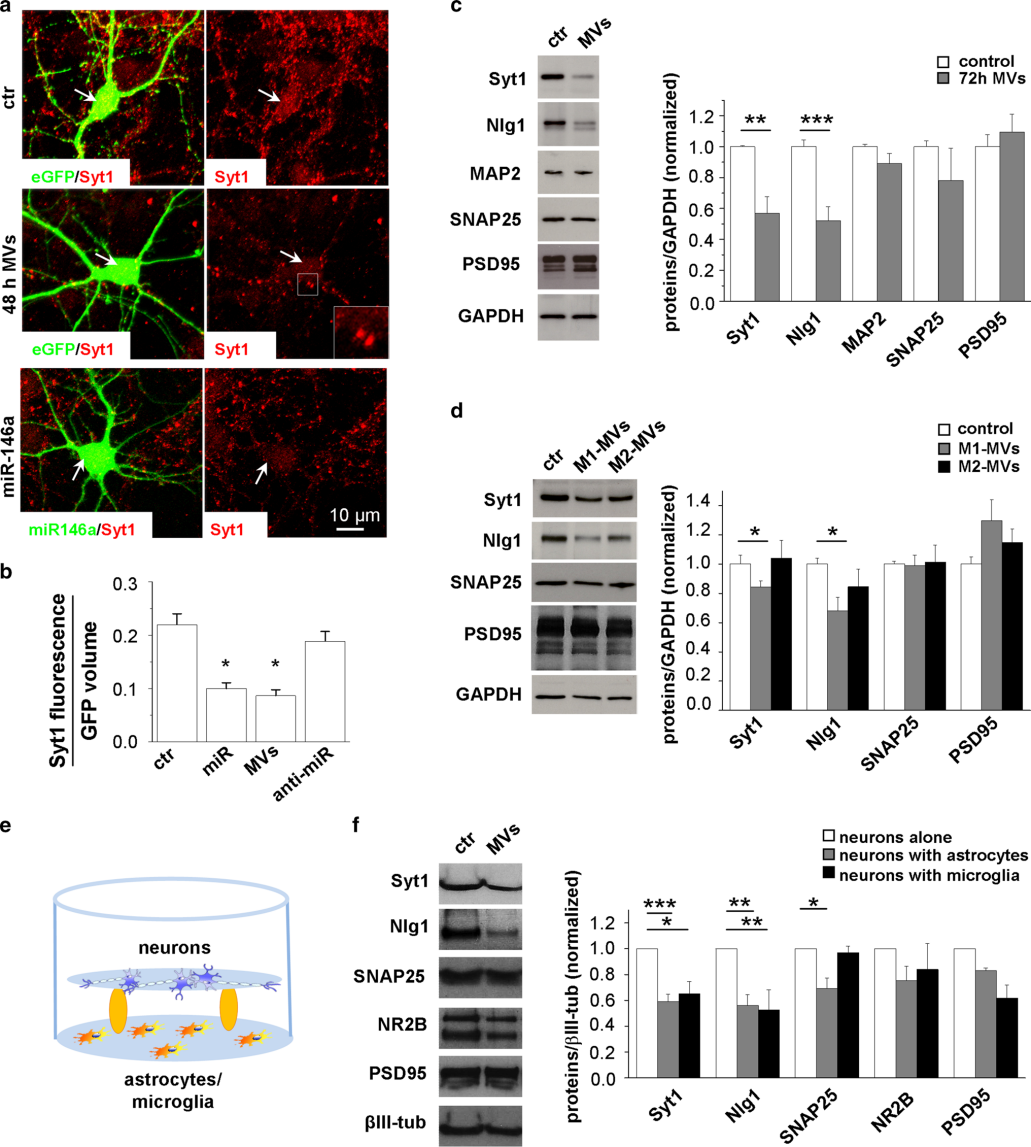
Decreased Syt1 and Nlg1 expression in neurons exposed to miR-146a-5p-enriched MVs secreted from inflammatory glia. **a** Representative pictures of Syt1 staining (red) in GFP-transfected neurons maintained in resting conditions (ctr) or exposed to MVs from LPS-treated astrocytes for 48 h (48 h-MVs) and in miR-146a-5p-GFP (BlockIt plasmid) transfected neurons. The structure of the cell body and proximal dendrites, where Syt1 fluorescence is measured, is revealed by eGFP. Arrows point to Syt1 staining in the cell bodies while examples of Syt1 positive pre-synaptic puncta are highlighted by a white box and shown at higher magnification in the inset. Scale bar 10 μm. **b** The histogram shows quantitative analysis of Syt1 fluorescence in the somatodendritic region of GFP-transfected neurons (ctr), miR-146a-5p-GFP transfected neurons (miR), GFP-transfected neurons exposed for 48 h to LPS-treated MVs (MVs) or from anti-miR-146a-5p-treated astrocytes (anti-miR). ANOVA Holm–Sidak multi-comparison test *P* = 0.05; number of neurons: ctr = 20, miR = 15, MVs = 20, anti-miR = 16, *n* = 3. **c** WB analysis of hippocampal neurons showing downregulation of Syt1 and Nlg1 but not MAP2, SNAP25 and PDS95 after 72 h treatment with MVs secreted from LPS-treated astrocytes. GAPDH is used as loading control. Unpaired Student’s *t* test: Syt1 *P* = 0.008, Nlg1 *P* = 0.001, MAP2 *P* = 0.342, SNAP25 *P* = 0.343, PSD95 *P* = 0.519; *n* = 3. **d** WB analysis of Syt1, Nlg1 SNAP25 and PSD95 in control neurons and neurons exposed to miR146-enriched MVs derived from inflammatory microglia (M1-MVs) or miR-146 depleted MVs derived from pro-regenerative microglia (M2-MVs) for 72 h. One-way ANOVA, Dunn’s method comparison test: Syt1 *P* < 0.05, Nlg1 *P* < 0.05, SNAP25 *P* = 0.676, PSD95 *P* = 0.2; *n* = 3. **e** Schematic representation neurons indirectly co-cultured with inflammatory astrocytes or microglia. **f** WB analysis of Syt1, Nlg1, NR2B and PSD95 in control neurons and neurons indirectly co-cultured with LPS-treated astrocytes or inflammatory microglia for 72 h. Unpaired Student’s *t* test for astrocyte coculture: Syt1 *P* < 0.001, Nlg1 *P* = 0.003, SNAP25 *P* = 0.011, PSD95 *P* = 1.000; *n* = 3 and Unpaired Student’s *t* test for microglia coculture: Syt1 *P* = 0.05, Nlg1 *P* = 0.007, SNAP25 *P* = 0.690, PSD95 *P* = 0.097; *n* = 3

To investigate whether MVs transfer bioactive miR-146a-5p to neurons, we first analysed immunoreactivity for Syt1, a validated miR-146a-5p target, in the soma and proximal dendrites of eGFP-transfected neurons. Astrocyte-derived MVs reduced by 50% Syt1 immunostaining 48 h after MV administration to neurons, similarly to direct miR-146a-5p transfection, and the MV effect was rescued by transfection of donor glia with anti-miR-146a-5p (Fig. 3a, b). The analysis was performed in the somatodendritic region because at this location Syt1 not incorporated into synapses is degraded at higher rate [19], and therefore early changes in Syt1 protein levels can be detected in this compartment. Accordingly, 48 h after MV addition, presynaptic terminals still displayed intense Syt1 fluorescence (Fig. 3a, arrowhead) and no change of total Syt1 protein levels was detected by western blot analysis (not shown). However, upon a longer treatment (72 h, with three MV additions, one every 24 h) MVs caused a significant decrease in expression of both Syt1 and Nlg1, another validated miR-146a-5p target (Fig. 3c). Inhibition of ATP-induced MV production by the purinergic antagonist oxidized-ATP (oATP) [7] prevented Nlg1 downregulation in receiving neurons (Fig. S4), confirming that ATP exacerbates the action of MVs. The impact of MVs-associated miR-146a-5p on Syt1 and Nlg1 expression was further explored in neurons exposed to miR-146a-5p-enriched or miR-146a-5p-depleted MVs derived from M1 and M2 microglia (added freshly to neurons every 24 h for 72 h). As expected, MVs released from M1 microglia, but not MVs produced from M2 cells, decreased protein levels of Syt1 and Nlg1 (Fig. 3d). Prolonged exposure to inflammatory MVs did not alter the expression of genes not predicted to be targeted by miR-146a-5p (SNAP25, PSD95, and MAP2), indicating the absence of synaptic or neuronal damage (Fig. 3c, d). Analysis of cell viability revealed similar percentage of calcein-positive and propidium iodide negative viable neurons in control and MVs-treated cultures (% of calcein^+^/PI^−^ neurons/overall nuclei: ctr = 69.32 ± 1.87; MVs = 81.83 ± 1.95; number of neurons: ctr = 355, MVs = 326; *n* = 3) further excluding neurotoxic effects.

Taken together, these data indicate that MVs secreted from inflammatory glial cells deliver bioactive miR-146a-5p to neurons and modulate the expression of its synaptic targets according to their miR-146a-5p cargo.

Next we investigated whether transfer of miR-146a-5p via MVs might occur into neurons, when indirectly co-cultured with inflammatory astrocytes or microglia (Fig. 3e), mimicking a more physiological condition. Western blot analysis showed a significant decrease in Nlg1 and Syt1 in neurons maintained with either inflammatory microglia (at a 2:1 ratio) or astrocytes (at a 1:2 ratio) for 72 h compared to neurons cultured alone (Fig. 3f). This suggests that reactive microglia and astrocytes deliver miR-146a-5p-storing MVs to neurons in biologically relevant quantities. Of note, protein levels of SNAP25 were also reduced in neurons co-cultured with reactive astrocytes, revealing the possible contribution of other secretory molecules not associated to EVs to synaptic gene silencing. Moreover, protein levels of PSD95, a target gene of miR-125-5p, a miRNA upregulated in exosomes released from inflammatory microglia (Table 1), tended to decrease in neurons cultured with inflammatory microglia, suggesting a possible role for the exosome-associated miRNA in the control of synaptic genes.

### Inflammatory MVs affect synaptic stability via Nlg1 downregulation

**Fig. 4:**
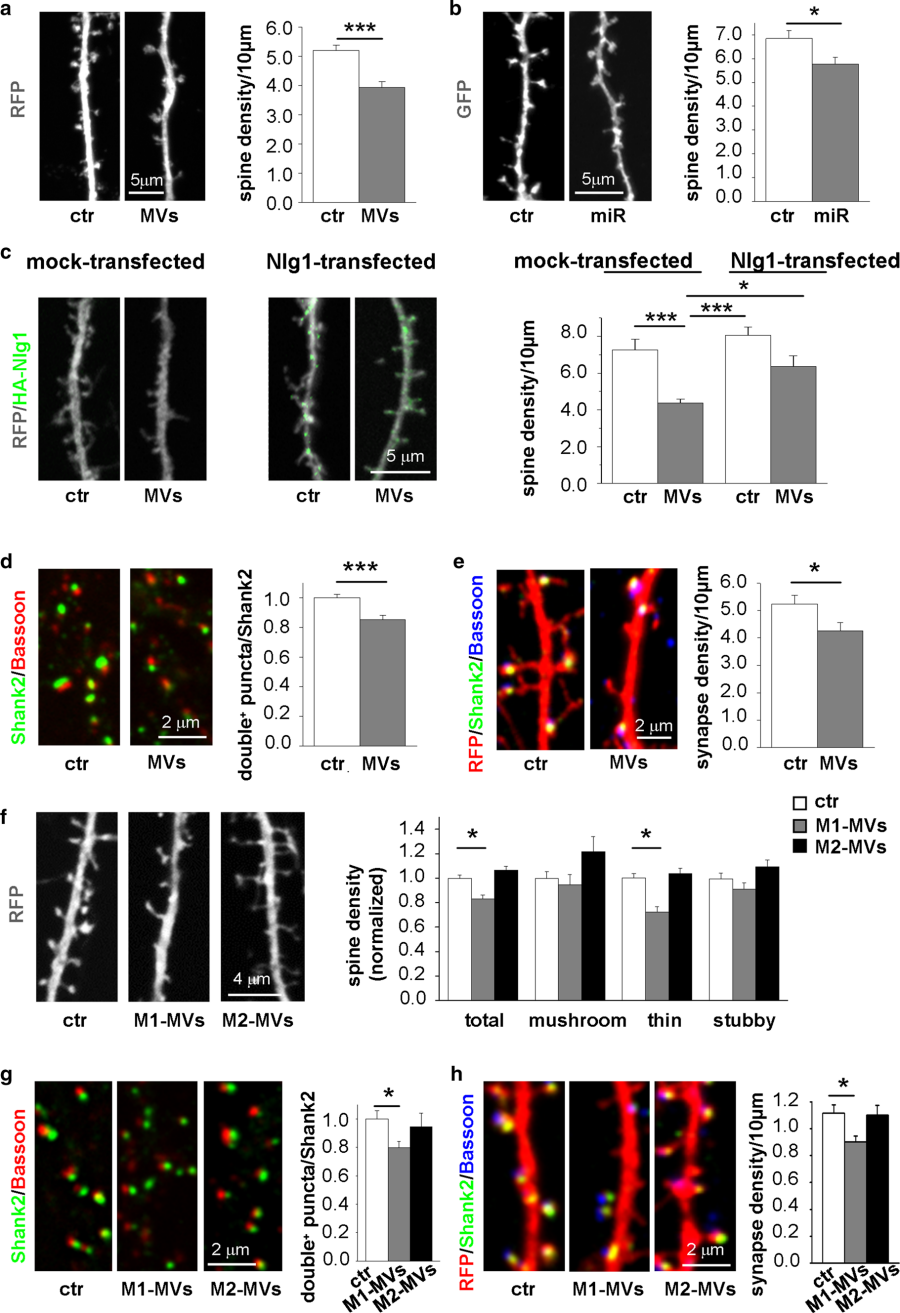
Decreased density of dendritic spines and synapses in neurons exposed to MVs secreted from inflammatory astrocytes and microglia. **a** Analysis of spine density in 17-day-old RFP-positive neurons (ctr), and neurons exposed for 72 h to MVs derived from LPS-treated astrocytes (MVs). Unpaired Student’s *t* test *P* ≤ 0.001; number of neurons: ctr = 70, MVs = 71, *n* = 3. Scale bar 5 μm. **b** Analysis of spine density in control GFP-transfected neurons (ctr) and miR-146a-5p-GFP transfected neurons (miR). Mann–Whitney rank sum test: *P* = 0.013; number of neurons: ctr = 42, MVs = 42, *n* = 3. Scale bar 5 μm. **c** Representative fluorescence pictures of RFP-positive neurons, co-transfected with hemagglutinin (HA)-tagged Nlg1 (green) or mock-transfected, maintained in control conditions or exposed to MVs from LPS-treated astrocytes. The histograms show corresponding dendritic spine density. ANOVA, Tukey multiple comparison test: *P* ≤ 0.001; number of neurons: ctr = 26, MVs = 27, Nlg1 = 28, NLG1 + MVs = 24, *n* = 3. Scale bar 5 μm. **d** Staining for the presynaptic marker Bassoon (red), and the postsynaptic protein Shank2 (green) in control neurons and neurons treated with astrocytic MVs. The histograms show the percentage of juxtaposed pre- and post-synaptic terminals (Bassoon & Shank2 co-localizing puncta) relative to the total post-synaptic puncta (Shank2 puncta). Unpaired Student’s *t* test: *P* ≤ 0.001; number of fields: ctr = 78, MVs = 82, *n* = 3. Scale bar 2 μm. **e** Control and MVs-treated RFP-positive neurons (red) stained for Bassoon (blue) and Shank2 (green). Histograms show corresponding density of synaptic contacts. Unpaired Student’s *t* test: *P* = 0.028; number of neurons: ctr = 22, MVs = 22, *n* = 3. Scale bar: 2 μm. **f** Analysis of total spine density in untreated RFP-positive neurons (ctr) or cells incubated with MVs derived from inflammatory (M1-MVs) or pro-regenerative (M2-MVs) microglia. ANOVA, Tukey multiple comparison test: *P* ≤ 0.05, number of neurons for total spine density: ctr = 62, M1-MVs = 60, M2-MVs = 62; *P* = 0.293, number of neurons for mushroom spine density: ctr = 62, M1-MVs = 60, M2-MVs = 62; *P* ≤ 0.05, number of neurons for thin spine density: ctr = 62, M1-MVs = 60, M2-MVs = 62; *P* = 0.069, number of neurons for stubby spine density: ctr = 62, M1-MVs = 60, M2-MVs = 62; *n* = 3. Scale bar 4 μm. The decrease in the number of total protrusions in M1-MVs-treated neurons is mostly due to a decrease in immature thin spines. **g** Staining for Bassoon (red) and Shank2 (green) in control neurons and neurons treated with M1-MVs or M2-MVs. The histograms show the percentage of Bassoon and Shank2 co-localizing puncta relative to postsynaptic puncta. ANOVA, Tukey Multiple comparison test: *P* ≤ 0.05, number of fields: ctr = 33, M1-MVs = 33, M2-MVs = 33; *n* = 3. Scale bar 2 μm. **h** RFP-positive neurons stained for Bassoon (blue) and Shank2 (green) under resting condition or after incubation with M1-MVs or M2-MVs. Histograms show corresponding density of synaptic contacts. ANOVA, Tukey Multiple comparison test: *P* ≤ 0.05, number of neurons: ctr = 48, M1-MVs = 48, M2-MVs = 40; *n* = 3. Scale bar 2 μm

Nlg1 is a postsynaptic protein selectively expressed at excitatory synapses which is known to regulate spine formation and synapse stability through binding to presynaptic neurexins [16, 70]. Thus, Nlg1 downregulation might affect dendritic spine and synapse density in neurons receiving inflammatory EVs. We tested this hypothesis in neurons transfected with RFP, to delineate dendritic spine morphology. 72 h incubation with MVs derived from inflammatory astrocytes caused a significant decrease in spine density (Fig. 4a), mimicking the reduction induced by transfection with miR-146a-5p-GFP (Fig. 4b). To prove the involvement of Nlg1 downregulation in dendritic spine destabilization, we transfected neurons with a miR-146a-5p-resistant Nlg1, lacking 3′-UTR sequence. Rescue of Nlg1 expression prevented the decrease in spine density caused by inflammatory MVs (Fig. 4c).

Immunocytochemical staining for the presynaptic active zone marker Bassoon and the postsynaptic density marker Shank2 showed a significant decrease in the percentage of juxtaposed pre- and postsynaptic terminals (Fig. 4d) and in synaptic density (Fig. 4e) in neurons receiving inflammatory MVs. Thus, loss of dendritic spines is accompanied by altered synaptic density.

### MVs secreted from inflammatory but not pro-regenerative microglia reduce spine and synaptic density

Dendritic spine destabilization in response to miR-146-enriched MVs was confirmed in neurons treated with MVs released from inflammatory microglia (M1-MVs), where loss of immature (thin) spines mostly accounted for reduced spine density (Fig. 4f). Similarly to MVs produced from reactive astrocytes, M1-MVs also decreased the fraction (Fig. 4g) and density (Fig. 4h) of juxtaposed pre- and postsynaptic terminals. Conversely, MVs secreted from M2 microglia, depleted in miR-146a-5p, did not cause spine loss and rather tended to increase the density of mature (mushroom) spines (Fig. 4f). In addition, they had no effect on the fraction of juxtaposed pre- and postsynaptic terminals (Fig. 4g) and the density of synaptic contacts (Fig. 4h).

**Fig. 5:**
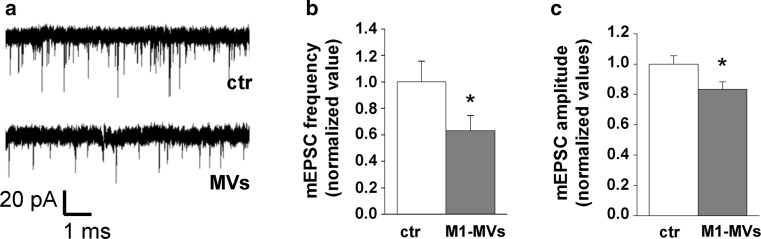
Inflammatory MVs decrease frequency and amplitude of mEPSCs. **a** Representative traces of mEPSCs from control and MVs-treated hippocampal cultures. **b** Summary histogram showing the mean frequency of mEPSCs from control and MVs-treated neurons (*P* = 0.024 Mann–Whitney rank sum test) **c**. Summary histogram showing the mean amplitudes of mEPSCs from control and MVs-treated neurons (number of cells: ctr = 33, MVs = 31; *P* = 0.036 Mann–Whitney rank sum test)

To evaluate the impact of inflammatory MVs on synaptic transmission, we analysed miniature excitatory postsynaptic currents (mEPSCs) in 14-day-old neurons (Fig. 5a). Inflammatory MVs caused a significant decrease in mEPSC frequency (Fig. 5b) and amplitude (Fig. 5c), indicating a decrease in synaptic strength.

### Chronic exposure to inflammatory EVs induces loss of dendritic spine in vivo

**Fig. 6:**
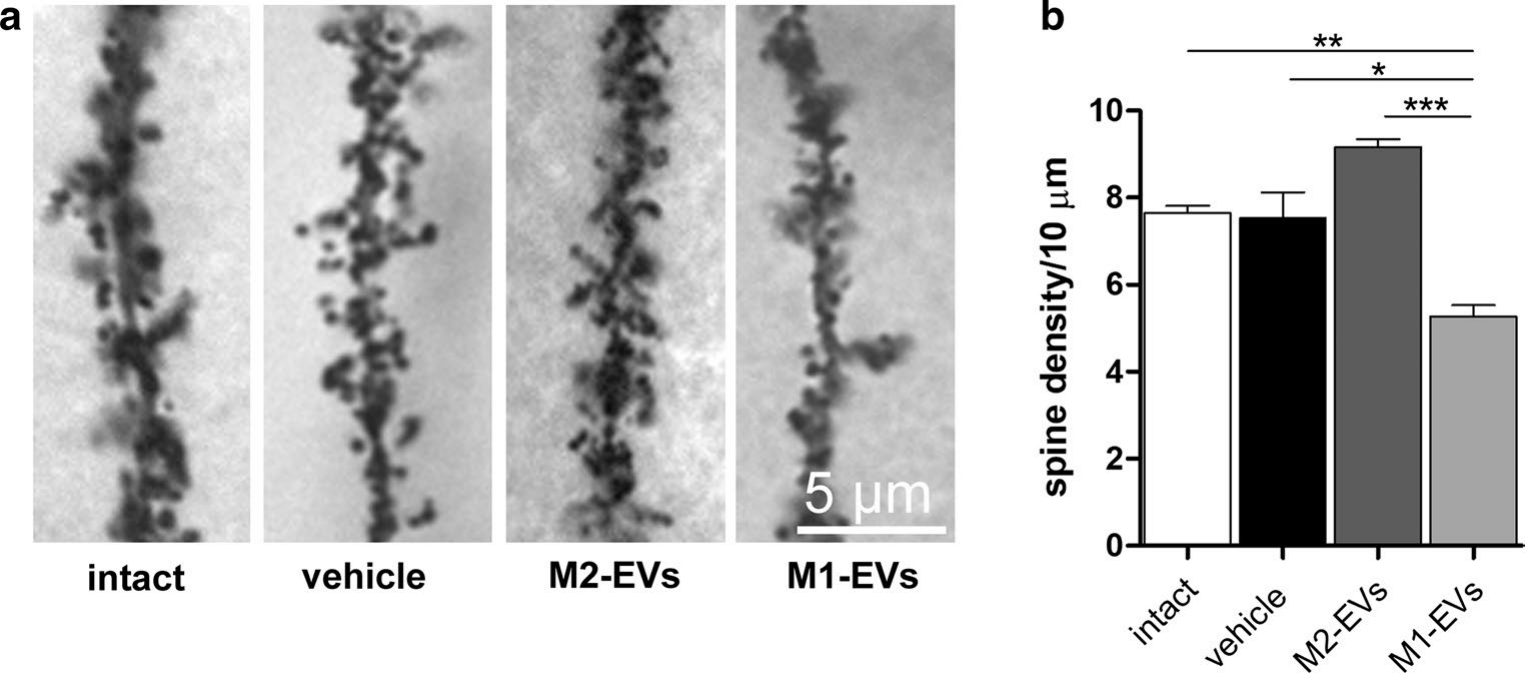
Exposure to M1-EVs, but not M2-EVs, reduces spine density in CA1 neurons. **a** Representative pictures of secondary dendrites of CA1 neurons in intact mice (contralateral hippocampus), vehicle-injected mice or upon treatment with either M2- or M1-EVs derived from rat microglia. **b** Quantification of spine densities. ANOVA, Bonferroni multiple comparison test, main effect of treatment: *P* = 0.0004; total length analysed: 2778 μm intact, 2694 μm vehicle, 3124 μm M2-EVs, 2794 μm M1-EVs, 90 dendrites for group, *n* = 3 mice. Scale bar 5 μm

To validate our findings in vivo, M1**-**EVs derived from inflammatory rat microglia enriched in miR-146a-5p were administered to mice by chronic delivery (4 days) with pre-filled Alzet mini-pumps, implanted into the CA1 region of the hippocampus. Quantitative analysis, performed on 100-μm-thick brain sections stained by Golgi method, showed a decrease of ~ 30% in spine density compared to intact control, vehicle- and M2-EVs-injected mice (Fig. 6a, b). This result confirms in vivo that miR-146a-5p-enriched EVs released from inflammatory microglia promote dendritic spine loss, likely through downregulation of Nlg1, which is a validated miR-146a-5p target in both rat and mouse brain.

### EVs fuse with neurons to deliver their miRNA cargo

**Fig. 7:**
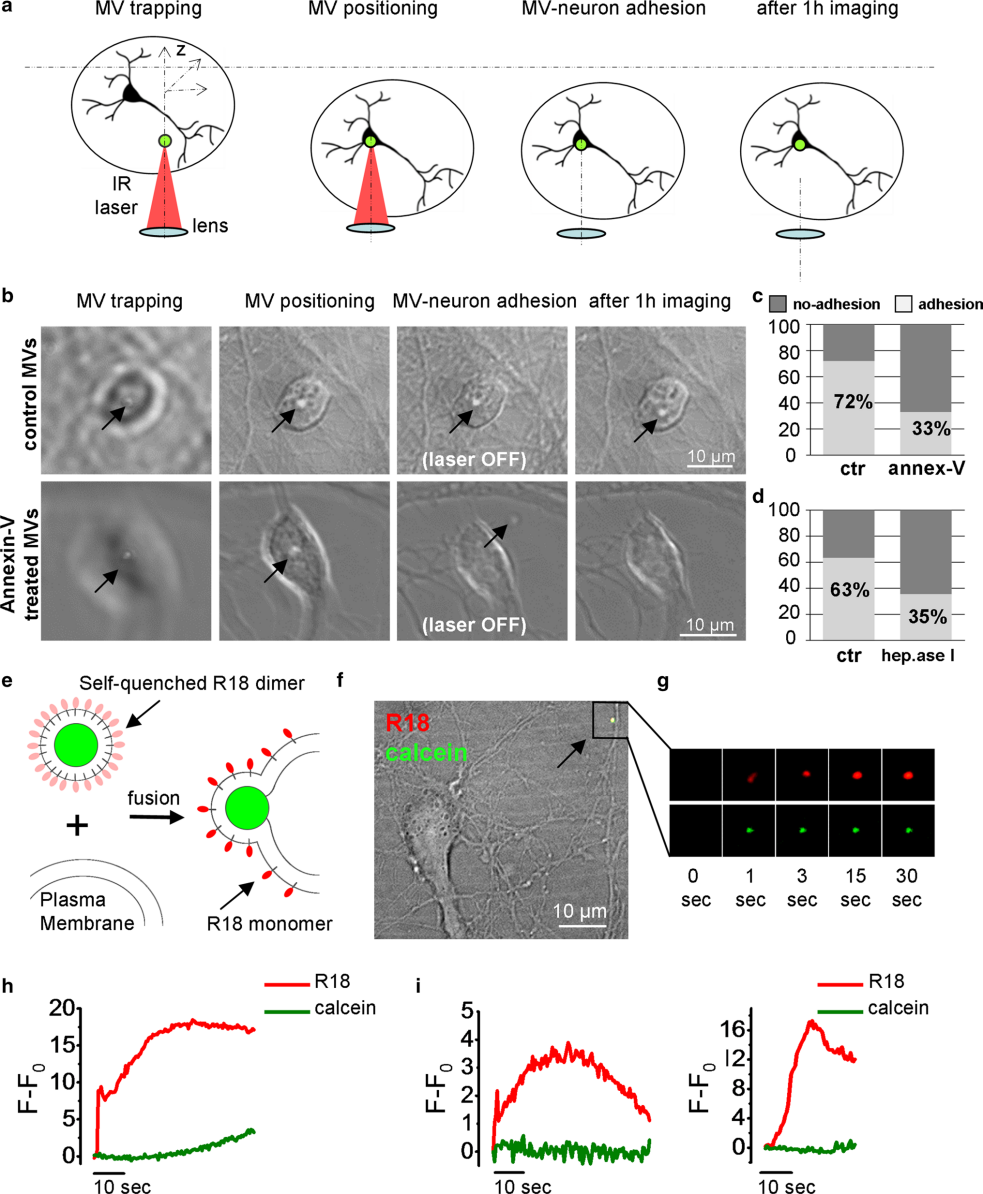
Transient fusion of glia MVs with the neuron PM. **a** Schematic representation of MV delivery to neurons by optical tweezers. MV is first trapped above the neurons by the IR laser tweezers (left), then the stage is moved in plane (XY) and the objective/trap is moved axially (Z) to set the MV in contact with the neuron (middle). The trapping laser is switched off to check whether MV adheres to the neuron membrane (right). **b** Sequence of phase-contrast images showing examples of control (top) and Annexin-V-treated (bottom) MVs driven to a neuron following the procedure described in **a**. **c**, **d** Histograms show the percentage of MVs adhesion to neurons after treatment of MVs with annexin-V (**c**) and of neuron with heparitinase I (**d**). **e** Schematic representation of R18 (red) dequenching upon fusion of calcein-loaded (green) MVs stained with self-quenching concentration of the dye. Persistence of calcein fluorescence indicate the opening of a pore non-permeable to the dye. **f** Merged bright field and fluorescence image of a calcein-loaded MVs stained with self-quenching concentration of R18 approaching to the surface of neuronal processes (arrow). **g** Time lapse images of the MV during contact with the neuron PM. **h** Corresponding temporal plots of calcein and R18 fluorescence. **i** Representative temporal plots of other fusion events between MVs and neurons

Next we asked how MVs transfer their miRNA cargo to neurons. EV–neuron interactions were analysed by delivering single MVs to neurons by optical tweezers and monitoring MV–neuron contact by time lapse microscopy [59]. After MV addition to neurons, MVs still suspended in the medium were trapped by the IR laser tweezers and positioned on neuron cell bodies in spatially controlled manner. The trapping laser was switched off after 30 s to prove MV–cell interaction (Fig. 7a). A large fraction of astrocyte-derived MVs adhered to the cell body of neurons (~ 74% *n* = 47) and more than half of microglial MVs was able to bind to neuronal surface (~ 53% *n* = 30). Cloaking phosphatidyl-serine (PS) residues on MV surface with annexin-V decreased MV adhesion by 54% (number of ctr = 18, annexin-treated MVs = 15) proving that interaction of vesicular PS with corresponding neuronal receptor mediates contact of approximately half MVs (Fig. 7b, c). MV–neuron contact was also inhibited by 44% when neurons were treated with heparitinase I, which cleaves HS chain of transmembrane proteoglycans (syndecans) (number of ctr = 41, heparitinase-treated neurons receiving MVs = 31) (Fig. 7d). This finding revealed that neuronal syndecans contribute via their HS chain to MV adhesion. After contact with neuron cell bodies, MVs showed little displacements around the place of delivery and remained attached to the neuron surface for up to 1–2 h of recording (Fig. 7b). Importantly, none of the MVs attached to neurons disappeared from the observation field, thus excluding rapid MV uptake or full fusion with the neuron plasma membrane (PM). Based on these findings we next investigated whether EV–neuron contact may be accompanied by transient fusion of the MVs with the PM. By spinning disk confocal microscopy we imaged the contact between neurons and MVs, pre-loaded with calcein, a fluorescent probe (green) that gets trapped in the MV lumen [1] and with self-quenching concentrations of the lipophilic membrane dye R18 (red) [48] (Fig. 7e). Briefly, double-stained MVs were added to neuronal medium and their movement by gravity towards neurons in the microscope field was tracked using green calcein fluorescence. When single MVs approached the neuron membrane, calcein (green) and R18 (red) fluorescence images were collected at 2 scan/s along with bright field to track dynamics of MV–neuron interaction. An example of MV approaching to the surface of a neurite is shown in Fig. 7f, g. Temporal plot of R18 fluorescence intensity revealed increases in R18 signal at the site of contacts (Fig. 7h, i), indicating dequenching of the dye upon fusion and dilution into the neuron PM. R18 dequenching was not accompanied by a decrease in calcein fluorescence, suggesting that fusion between MVs and neurons led to the opening of a transient pore, non-permeable to calcein.

## Discussion

Our study reveals a previously unrecognized mechanism behind loss of excitatory synapses during brain inflammation. We show that miR-146a-5p, which represses translation of presynaptic Syt1 and postsynaptic Nlg1, is upregulated in EVs secreted from reactive microglia either exposed to inflammatory or degenerative stimuli, and is delivered to neurons upon EV fusion with the plasma membrane. Notably, protein levels of Syt1 and Nlg1 are decreased, the density of dendritic spines and excitatory synapses is reduced, and spontaneous miniature synaptic currents are diminished in receiving neurons, indicating important synaptic alterations.

Our previous studies have shown that microglia-derived MVs affect the presynaptic compartment causing an excitation–inhibition unbalance. MVs acutely enhance glutamate release while suppress spontaneous GABA transmission in both hippocampal cultures [62] and in the visual cortex [1] and their action is due to the lipid components of MVs. This has been clearly proved at GABAergic terminals, where MVs-associated endocannabinoids stimulate cannabinoid receptor type 1 (CB1) and rapidly decrease spontaneous GABA release [29]. Importantly, presynaptic changes caused by microglial MVs are independent of the phenotype of donor microglia (unstimulated, pro-inflammatory or pro-regenerative cells) [1], suggesting that bioactive lipids secreted in association with MVs may contribute to homeostatic regulation of neurotransmission, in the absence of inflammation and microglia reaction.

In this study, we have identified miRNAs as additional active components of inflammatory MVs that are responsible for a delayed destabilization of excitatory synapses and decreased transmission, which selectively occur during chronic inflammation.

### EVs transfer their miRNA cargo to neurons

We have provided formal proof for EV-mediated shuttling of miRNAs between reactive glia and neurons and consequent downregulation of their synaptic targets in a number of ways: (1) monitoring neuronal levels of cel-miR-39, a *C. elegans* miRNA not present in rat neurons, with a *Renilla*-based specific reporter indicated that MVs exogenously expressing the cel-miR-39, secreted from cel-miR-39 transfected astrocytes, deliver their miRNA cargo to the cytoplasm of neurons; (2) monitoring miR-146a-5p, a glia-specific miRNA upregulated in EVs produced by inflammatory glia, showed that miR-146a-5p levels increase in neurons transfected with a specific *Renilla* reporter in response to inflammatory EVs but not EVs released from anti-miR-146a-5p-treated cells; (3) glia-to-neuron transfer of miR-146a-5p did not occur when EV–neuron contact was prevented by cloaking vesicular PS, a key signal for EV recognition on neurons.

### MVs-associated miR-146a-5p causes synaptic alterations through Nlg1 downregulation

The main accomplishment of our study is the demonstration that exchange of miR-146a-5p (and possibly other miRNAs) between inflammatory microglia and neurons via EVs causes important alterations of synaptic structure and function, independent of neuronal damage.

We have shown that transfer of miR-146a-5p via EVs is sufficient to decrease Syt1 and Nlg1 expression, to reduce dendritic spine in vitro and in the CA1 region of the hippocampus and to decrease synapse density and strength in cultured neurons, revealing that this miRNA hampers proper synapse stability and function. This evidence arose from several observations: (1) reduced Syt1 and Nlg1 expression in neurons incubated with miR146a-enriched MVs (derived from inflammatory microglia or astrocytes), but not miR-146a-5p-depleted MVs (released from pro-regenerative microglia) or MVs storing inactive miR-146a-5p (produced by astrocytes treated with a miR-146a-5p inhibitor); (2) reduced spine and synapse density in neurons exposed to miR146a-enriched MVs, derived from either reactive microglia or astrocytes, not occurring when neurons were exposed to miR146a-depleted MVs, derived from IL4-treated microglia; (3) decreased Syt1 immunoreactivity and decreased spine density in neurons transfected with miR-146a-5p. We also demonstrated that miR-146a-5p shuttling destabilizes dendritic spines via Nlg1 downregulation, as indicated by rescue of dendritic spine density in neurons expressing a miR-146-insensitive Nlg1, treated with inflammatory MVs.

Other miRNAs targeting synaptic genes are enriched in MVs produced by inflammatory microglia. Among them, miR-223, which targets the AMPA-selective glutamate receptor 2 (GluR2) and the NMDA glutamate receptor gluN2B [33] and miR-181a which regulates only gluN2B [66], a receptor essential to form LTP [8, 71]. We did not find significant changes in gluN2B protein expression in neurons co-cultured with inflammatory glial cells. However, the decreased amplitude of AMPA-mediated mEPSC suggests that miR-223 may indeed be involved in alteration of spontaneous excitatory transmission in MVs-treated neurons. Additional experiments are necessary to clarify whether MVs-associated miR-223 may alter GluR2 expression in neurons and whether synaptic dysfunctions may result from the combined action of multiple miRNAs.

Previous work has linked changes in miRNA expression and EV production to synaptic plasticity. Neuron depolarization has been shown to induce release of EVs containing high amounts of miRNAs, and miRNA elimination has been proposed to promote rapid translation of proteins required for synaptic plasticity [31]. Our study extends and complements these findings by showing that glia-to-neuron transfer of miR-146a-5p suppresses translation of a key dendritic protein, Nlg1, and decreases synaptic density and strength. These synaptic changes may represent a compensatory response to preserve neuronal circuit, and counteract acute hyperexcitability and excessive glutamatergic transmission, caused by inflammatory cytokines released by reactive glia. Consistent with the idea of a rescue mechanism, miR-146a-5p has well known immunosuppressive and anti-inflammatory functions [20, 35, 76]. Its silencing activity has recently been shown to decrease hyperexcitability-underlying seizures in a model of epileptogenesis, where gliosis and inflammatory cytokines are major pathogenic factors [34]. However, in this study we show that sustained exposure of neurons to miR-146a-5p-enriched MVs causes over-inhibition of synaptic function and excessive synapse destabilization, leading to pathological loss of synapses, not linked to neuron damage, which may underlie synaptic dysfunction occurring in chronic inflammation.

### EVs-mediated shuttling of miRNAs from microglia to neurons: possible mechanism at the bases of cognitive symptoms?

The enrichment of miRNAs targeting dendritic genes in EVs secreted from microglia under both inflammatory and neurodegenerative conditions suggests that these miRNAs maybe linked to common alteration of synaptic functions occurring in both inflammatory and degenerative contexts. Consistently, miR-146a-5p, miR-181a and miR-223 have been previously shown to be dysregulated in the CNS, plasma, and/or immune cells of MS patients [36] but also implicated in neurodegenerative diseases [22, 32, 37, 61].

Multiple sclerosis (MS) is a synaptic disorder characterized by chronic microglia activation and enhanced EV secretion [78] with early onset compared to other neurodegenerative diseases. Despite a quarter of MS patients suffer symptoms arising from neuronal dysfunction, such as episodic memory impairment, already at the first clinical episode and show hippocampal microstructural damage [57, 63] we still lack a full understanding of how synaptic alterations start and how they are linked to microglia activation. Here we show that miR-146a-5p, together with miR-181a and miR-223 are present in EVs isolated from the CSF of a group of MS patients. This evidence provides a possible link between microglia activation, enhanced EV production and cognitive symptoms in MS patients. Despite not attracting interest as specific MS biomarkers, we anticipate that analysis of miRNA expression in myeloid EVs isolated from CSF or plasma of single subjects might clarify whether miR-146a-5p, miR-223 and/or miR-181a content has power to reflect or predict the development of cognitive symptoms in MS as well as in other neuroinflammatory disorders. Besides being useful biomarkers of cognitive dysfunction, these miRNAs may also represent therapeutic targets for the improvement of cognitive symptoms in brain inflammatory diseases.

### Inflammatory MVs deliver their cargo to neurons through transient fusion with the PM

Despite recent advances in EV research, how RNA content of EVs is delivered to target cells and whether all EVs are capable to deliver their cargo is still largely unclear [75]. Using optical tweezers, a unique tool to finely position single EVs on target cells and to study EV-cell interaction individually [59], here we show that a relevant fraction of MVs derived from microglia or astrocytes adhere to neurons, and can therefore be potentially functional as communication vehicles. MVs are heterogeneous with respect to their adhesive properties. The exposure of PS, a known recognition signal for receiving cells [50], mediates binding of about half glial MVs to neurons. Similarly, heparan sulfate proteoglycans [14] on the surface of receiving neurons mediate the interaction with only a fraction of MVs, indicating that multiple molecules are likely to participate in MV–neuron contact.

Further work remains to be done to define the minimum number of inflammatory EVs required to alter miR-146a-5p levels in neurons and to suppress its target genes, a challenging goal that goes beyond the aim of the present study. Nevertheless, co-cultures experiments suggest that reactive glia deliver miR-146a-5p to neurons via MVs in biological relevant quantities. Indeed, both Syt1 and Nlg1 are significantly downregulated in neurons indirectly co-cultured with inflammatory microglia at a physiological ratio.

A prominent finding of our study is that glial EVs deliver their cargo to neurons by transient fusion. This is revealed by dynamic imaging of EVs stained within the lumen with calcein and in the membrane with the self-quenching lipophilic dye R18 [48]. MV fusion was proven by dequenching of R18 fluorescence at contact sites while calcein retention in MVs suggested the opening of small pores, not permeable to the cytosolic dye. To our knowledge, these data are the first direct evidence that glia-derived EVs fuse with the neuron plasma membrane. Importantly, EV fusion also occurs along neuronal processes, where synaptic activity is influenced by EVs [1, 62]. EV fusion allows direct retrieval of EV cargo in the cytosol of neurons, avoiding degradation in late endosome or lysosomes, where EVs may accumulate after endocytosis [13, 26, 28]. Such a mechanism would also overcome the issue of how miRNAs might be retrieved back from endocytic compartments. Fusion of EVs with the neuron membrane is consistent with previous studies showing that EVs of microglia and neuron origin efficiently bind to the cell surface of neurons but are only rarely [13], if ever [29], internalized.

## Electronic supplementary material

Below is the link to the electronic supplementary material.

Characterization of microglia purity. (a-c) Representative images of primary microglia stained with the myeloid marker IB4 (green), the nuclear marker DAPI (blue) and the astrocyte marker GFAP (red) (a) or the oligodendrocyte progenitor marker NG2 (red) (b). (c) Pie chart shows the percentage of contaminating astrocytes and oligodendrocytes in the microglia culture. Values are mean ± SE. (TIFF 9551 kb)

MiRNA expression in EVs versus donor microglia. Scatter plot representations of miRNA expression in pooled EVs and unstimulated microglia. (TIFF 9571 kb)

MiRNA profiling in MVs and exosomes released from microglia with different activation state. a-c Venn diagrams of the numerical values for common and unique miRNAs present in MVs (yellow), exosomes (green) and parental microglia (blue) exposed to inflammatory (A), neurodegenerative (B) or pro-regenerative (C) stimuli. (TIFF 10102 kb)

Inhibition of ATP-induced MV production prevented Nlg1 downregulation in receiving neurons. WB analysis of Nlg1, MAP2 and VAMP2 levels in hippocampal neurons after 72 hrs treatment with MVs secreted from LPS-treated astrocytes, stimulated with ATP in the absence (ATP-MVs) or presence of o-ATP (oATP-MVs). GAPDH is used as loading control. One-way ANOVA, Holm-Sidak multi-comparison test: Nlg1 P < 0.05, MAP2 P = 0.533, VAMP2 P = 0.359. (TIFF 1688 kb)
