# Supplementary figures and images for: Glia-to-neuron transfer of miRNAs via extracellular vesicles: a new mechanism underlying inflammation-induced synaptic alterations
Source: Acta Neuropathol. 2018 Jan 4;135(4):529–50. doi: 10.1007/s00401-017-1803-x (PMC5978931; doi:10.1007/s00401-017-1803-x)

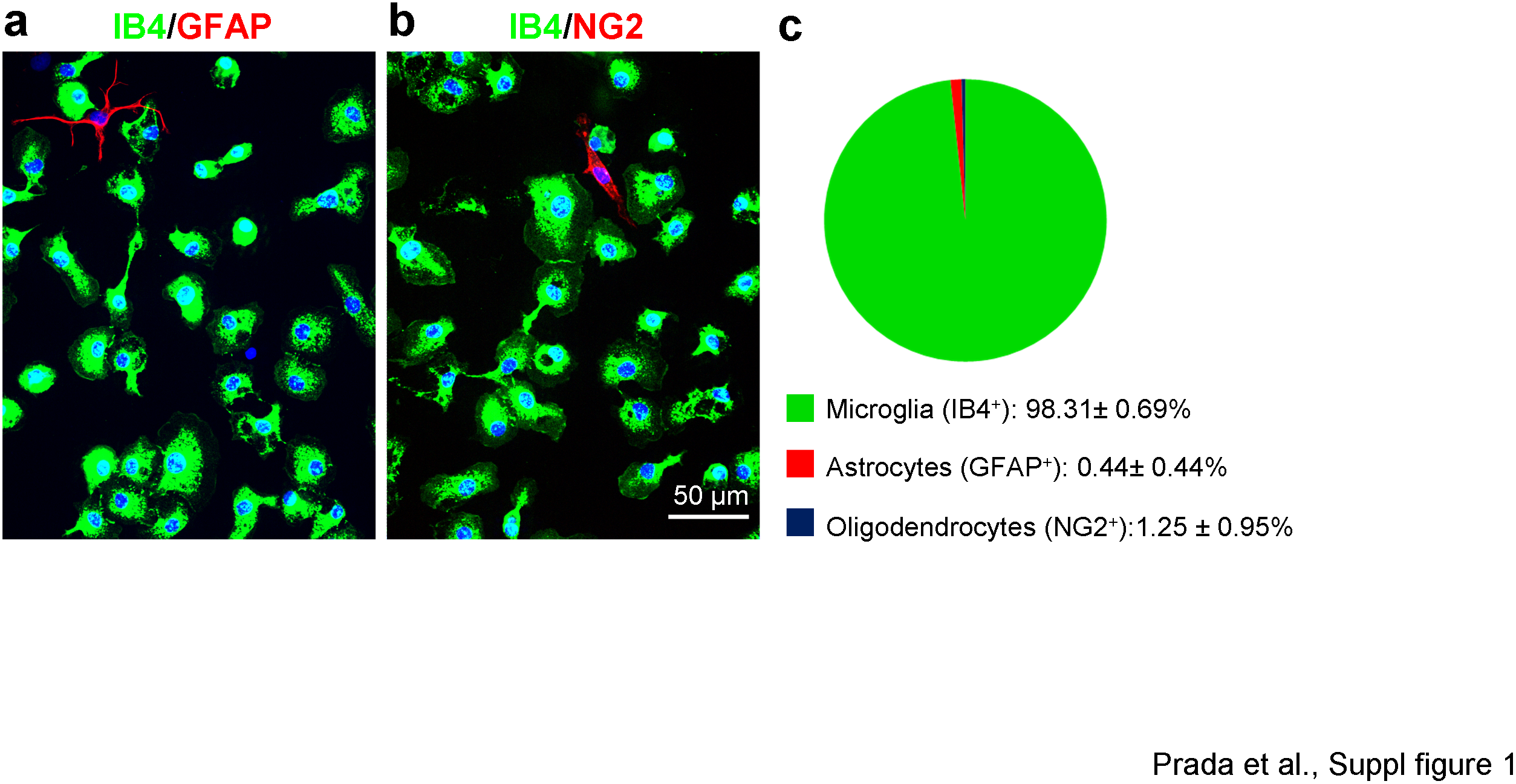

Supplement: Supplementary file 1 — Characterization of microglia purity. (a-c) Representative images of primary microglia stained with the myeloid marker IB4 (green), the nuclear marker DAPI (blue) and the astrocyte marker GFAP (red) (a) or the oligodendrocyte progenitor marker NG2 (red) (b). (c) Pie chart shows the percentage of contaminating astrocytes and oligodendrocytes in the microglia culture. Values are mean ± SE. (TIFF 9551 kb) [file 401_2017_1803_MOESM1_ESM.tif]

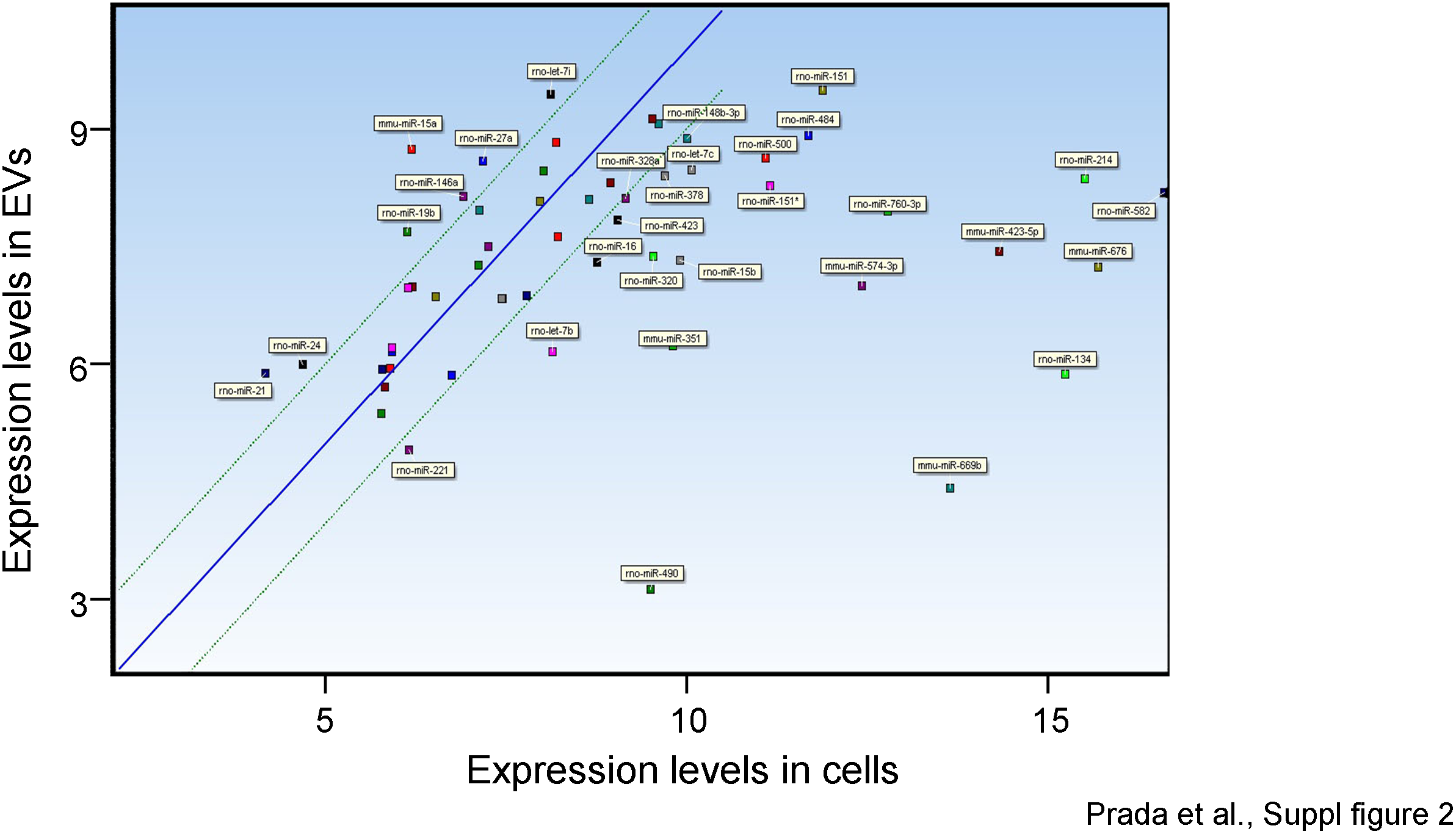

Supplement: Supplementary file 2 — MiRNA expression in EVs versus donor microglia. Scatter plot representations of miRNA expression in pooled EVs and unstimulated microglia. (TIFF 9571 kb) [file 401_2017_1803_MOESM2_ESM.tif]

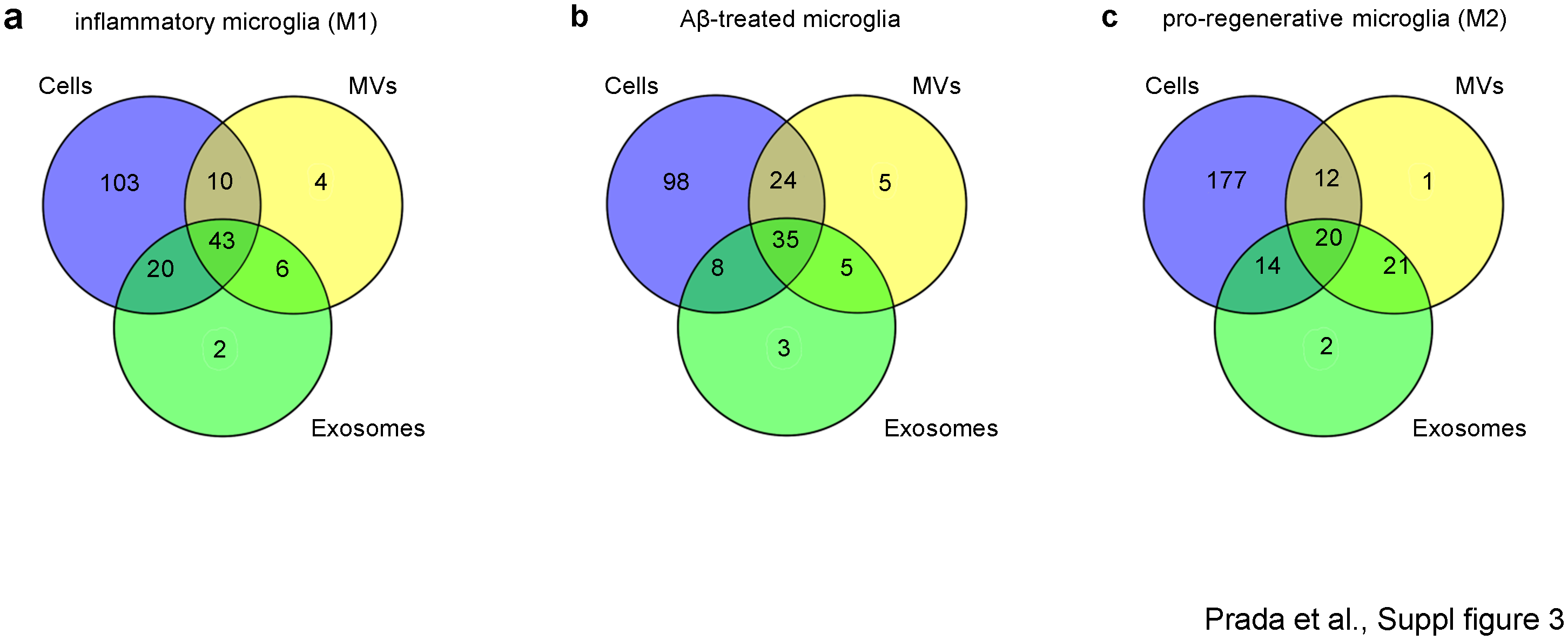

Supplement: Supplementary file 3 — MiRNA profiling in MVs and exosomes released from microglia with different activation state. a-c Venn diagrams of the numerical values for common and unique miRNAs present in MVs (yellow), exosomes (green) and parental microglia (blue) exposed to inflammatory (A), neurodegenerative (B) or pro-regenerative (C) stimuli. (TIFF 10102 kb) [file 401_2017_1803_MOESM3_ESM.tif]

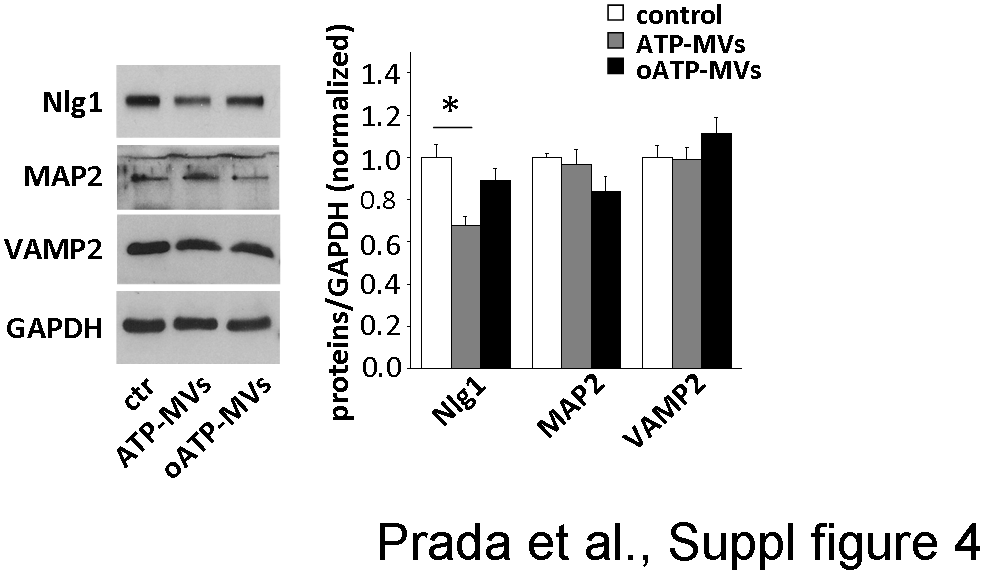

Supplement: Supplementary file 4 — Inhibition of ATP-induced MV production prevented Nlg1 downregulation in receiving neurons. WB analysis of Nlg1, MAP2 and VAMP2 levels in hippocampal neurons after 72 hrs treatment with MVs secreted from LPS-treated astrocytes, stimulated with ATP in the absence (ATP-MVs) or presence of o-ATP (oATP-MVs). GAPDH is used as loading control. One-way ANOVA, Holm-Sidak multi-comparison test: Nlg1 P < 0.05, MAP2 P = 0.533, VAMP2 P = 0.359. (TIFF 1688 kb) [file 401_2017_1803_MOESM4_ESM.tif]
